# Biexciton Blinking in CdSe-Based Quantum Dots

**DOI:** 10.1021/acs.jpclett.3c00437

**Published:** 2023-06-05

**Authors:** Sander J. W. Vonk, Freddy T. Rabouw

**Affiliations:** † Debye Institute for Nanomaterials Science, Utrecht University, Princetonplein 1, 3584 CC Utrecht, The Netherlands

## Abstract

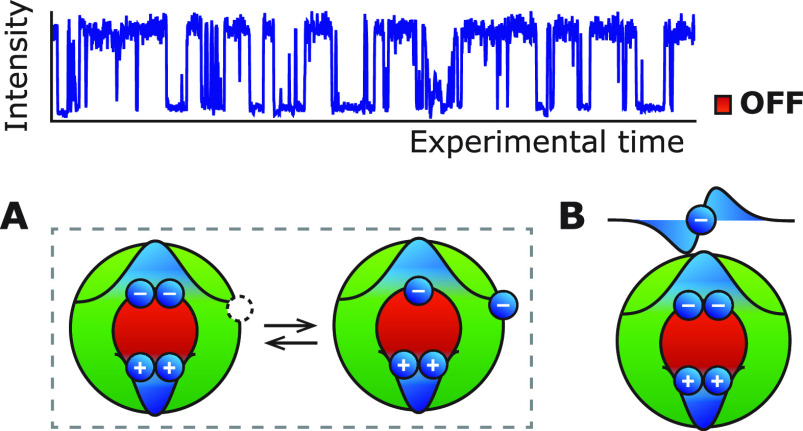

Experiments on single colloidal quantum dots (QDs) have revealed temporal fluctuations in the emission efficiency of the single-exciton state. These fluctuations, often termed “blinking”, are caused by opening/closing of charge-carrier traps and/or charging/discharging of the QD. In the regime of *strong* optical excitation, multiexciton states are formed. The emission efficiencies of multiexcitons are lower because of Auger processes, but a quantitative characterization is challenging. Here, we quantify fluctuations of the biexciton efficiency for single CdSe/CdS/ZnS core–shell QDs. We find that the biexciton efficiency “blinks” significantly. The additional electron due to charging of a QD accelerates Auger recombination by a factor of 2 compared to the neutral biexciton, while opening/closing of a charge-carrier trap leads to an increase of the nonradiative recombination rate by a factor of 4. To understand the fast rate of trap-assisted recombination, we propose a revised model for trap-assisted recombination based on reversible trapping. Finally, we discuss the implications of biexciton blinking for lasing applications.

Colloidal semiconductor quantum dots (QDs), with large absorption cross sections and high emission efficiencies, are a promising candidate for luminescent devices.^1−4^ However, temporal fluctuations of the emission efficiency (termed “blinking”) have been observed on the single-QD level, which lower the time-averaged emission efficiency.^5,6^ Significant research effort has been devoted to characterizing the intermittent quenching pathways for the single-exciton state. Charging/discharging, opening/closing of charge-carrier traps, and/or trapping of hot excitons may all contribute to these temporal fluctuations, observed for a wide range of semiconductor materials and shapes.^7−10^ However, understanding of the temporal fluctuations of multiexciton efficiencies is necessary to incorporate these materials in high-power devices such as lasers.

Different multiexciton emissions are difficult to distinguish in an experiment because of spectral and temporal overlap.^11,12^ In the regime of *weak* optical excitation, multiexciton emission is overwhelmed by a background of single-exciton emission, while *strong* optical excitation creates different multiexciton states simultaneously.^13^ Time-resolved single-QD experiments offer a solution: intensity-correlation measurements allow separating different multiexciton states by identifying their cascaded emission.^14−18^ Counterintuitively, while multiexciton emission is more likely at high optical excitation, the limit of weak optical excitation is necessary to best study biexciton emission.^11,19−21^ Such experiments are challenging because biexciton emission is relatively weak, but they ensure that biexciton emission is properly distinguished from both single-exciton and higher-multiexciton emissions. Previous studies using these intensity-correlation measurements challenged proposed quenching models of the single-exciton state and revealed enormous variations of multiexciton efficiencies within QDs from the same synthesis batch.^19,22,23^

In this Letter, we characterize temporal fluctuations of the biexciton efficiency for single CdSe/CdS/ZnS core–shell QDs at room temperature using intensity-correlation analysis in the regime of weak optical excitation. Our experiments confirm that the radiative decay rate of the biexciton is faster than that of the exciton by a factor 3.8 ± 0.3 (mean ± standard deviation averaged over 10 single QDs), consistent with statistical scaling^24^ and with previous experiments.^15^ We observe that the nonradiative decay rate fluctuates because of QD blinking. Opening of a charge-carrier trap increases the nonradiative decay rate of the biexciton by a factor of 3.9 ± 1.2 (mean ± standard deviation averaged over 5 single QDs) compared to the biexciton in the ON state. Additionally, we find that during intermittent charging of a QD, the addition of a charge carrier increases the nonradiative decay rate by a factor of 2.0 ± 0.2 (mean ± standard deviation averaged over 5 single QDs) compared to the neutral biexciton. Trap-induced quenching of the biexciton is more severe than expected based on a simple picture of charge-carrier trapping followed by nonradiative recombination. We thus propose a revised model for trap-assisted recombination where the initial step of charge-carrier trapping is reversible. Both opening/closing of traps and charging/discharging have implications for lasing from the exciton and the biexciton state, potentially increasing or decreasing the lasing threshold depending on the exact distribution of charged QDs, QDs with open traps, and regular QDs in a gain medium.

We first characterize the quenching pathways for exciton emission of our CdSe/CdS/ZnS core–shell sample with core radius of 1.9 nm, and nominally 8 monolayers of CdS and 2 monolayers ZnS (for details on the synthesis procedure, see Supporting Information Section S1). We perform pulsed-excitation experiments with a 405 nm diode laser operating at 2.5 MHz repetition rate (Supporting Information Section S2 for details on the experimental setup). Figure 1a,e shows representative parts of blinking traces for two different single QDs from the same synthesis batch (QD A in Figure 1a–c and QD B in Figure 1e–g; total acquisition time was on average 20 min per single-QD measurement). Both blinking traces show switching between two clearly resolved states: a high-intensity ON state and a low-intensity OFF state. For all single-QD measurements, we sometimes observe emission intensities in between the ON and OFF state and/or an emissive state with almost zero quantum yield. Intermediate emission intensities might be due to fast blinking events (or flickering) between the ON and OFF state, which leads to a time-averaged emission intensity of the ON and OFF state intensities.^24^ In the case of opening/closing of a charge-carrier trap (QD A, QD 6–10 in Supporting Information Section S6), spectral diffusion of the trap state and/or opening of a different trap state can also lead to intermediate and/or very low emission intensities. Here, we use the label “OFF” for the selected state that produces a low intensity compared to the ON state, whereas other studies sometimes use “gray” or “dim” depending on the exact intensity level. We analyze the excited-state dynamics of the selected emissive states by constructing fluorescence-lifetime–intensity distributions (FLIDs; Figure 1b,f) of a representative part of the experiment, which are two-dimensional histograms of photon counts and average lifetime per 5 ms time bins. Both QDs show a positive correlation between the emission intensity and the excited-state lifetime. To identify the mechanism of exciton quenching in the OFF state, we construct the time-averaged decay curves  of the ON (blue) and OFF (red) state in Figure 1c,g. In general, for any emissive state with radiative decay rate *k*_r_, nonradiative decay rate *k*_nr_, and total decay rate *k*_tot_ = *k*_r_ + *k*_nr_, such a time-averaged decay curve is given by
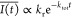
1where the amplitude *A* is proportional to the radiative decay rate *k*_r_.

**Figure 1:**
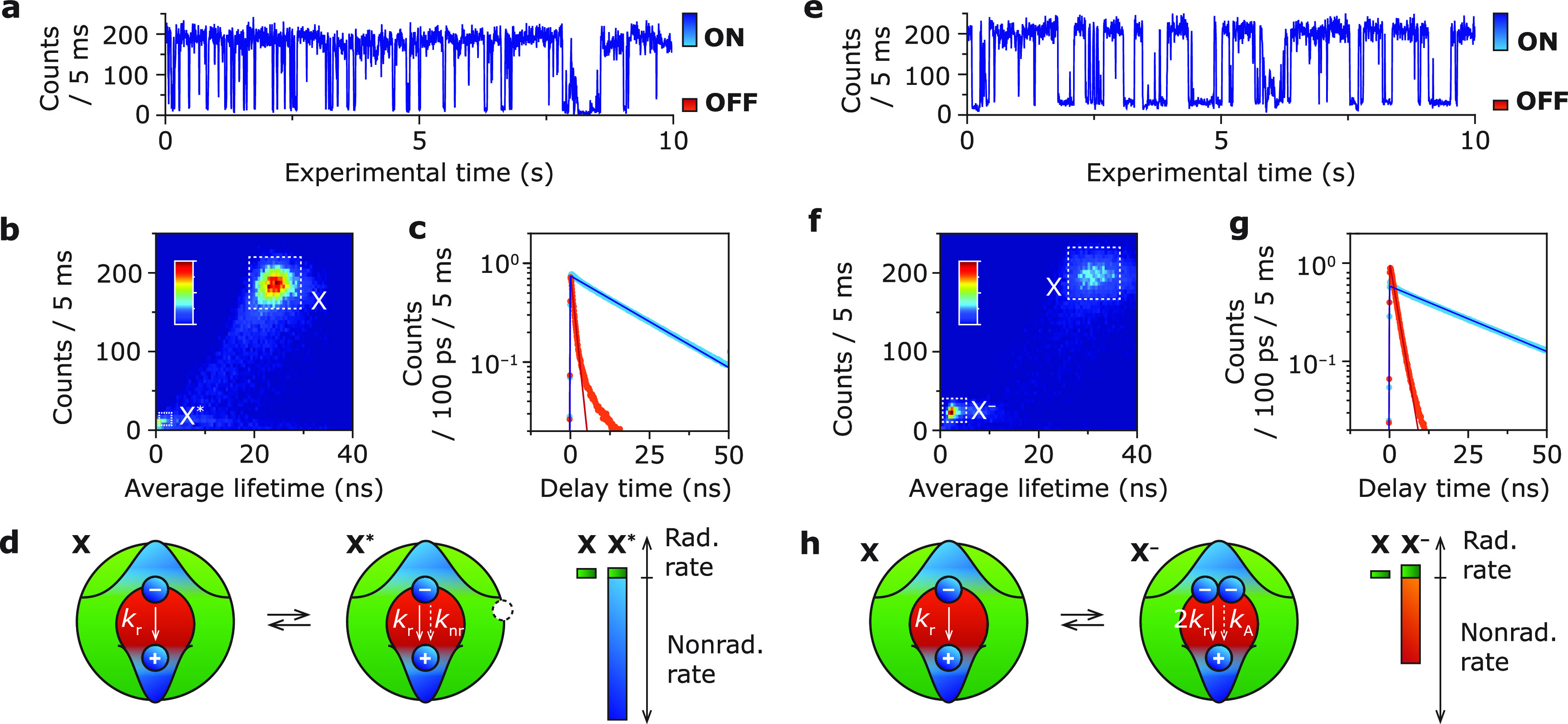
(a) Blinking trace of a single QD (QD A) showing switching between an ON (>170 counts per 5 ms) and OFF state (10–40 counts per 5 ms). (b) Fluorescence-lifetime–intensity distribution showing switching between a high-intensity/long-lifetime ON state and a low-intensity/short-lifetime OFF state. (c) Time-averaged decay curves of the ON (blue) and OFF (red) state. The amplitude of the OFF state is equal to that of the ON state indicating equal radiative rate in the ON and OFF state. (d) This is consistent with a quenching mechanism where charge-carrier trapping is in competition with radiative decay. The total decay rates are 0.04 ns^–1^ for the ON state X and 0.62 ns^–1^ for the OFF state X*. The inset shows the disentangled radiative (green) and nonradiative decay rate (blue) in the emissive states to scale. (e–h) Same as panels a–d, but for a different single QD (QD B) from the same synthesis batch. Here, the radiative decay rate of the OFF state (13–35 counts per 5 ms) is boosted by a factor of 2 compared to the ON state (>160 counts per 5 ms). This is consistent with charging, which doubles the number of radiative decay channels.^25^ The luminescence is quenched by Auger recombination in competition with radiative decay. The total decay rates are 0.03 ns^–1^ for the ON state X and 0.46 ns^–1^ for the OFF state X^–^. The inset shows the disentangled radiative (green) and nonradiative decay rate (red) in the emissive states to scale, which is indicative of charging introducing nonradiative Auger recombination in the OFF state.

The different OFF-state decay curves of the two QDs (Figure 1c,g) indicate different quenching mechanisms. We observe a difference between the amplitude ratio *A*_OFF_/*A*_ON_ of the ON and OFF state for the two different single-QD measurements (Figure 1c,g). The amplitude is approximately equal in the ON and the OFF state for QD A (Figure 1c) meaning that both emissive states have equal radiative rate (*k*_r,OFF_/*k*_r,ON_ = 1.1, accounting for the instrument response Supporting Information S3.1). This is consistent with intermittent opening and closing of a trap-assisted decay channel. With the trap open, radiative decay of the exciton is in competition with charge-carrier trapping.^8,26,27^ Previous studies showed that the ON state of single QDs has unity efficiency, meaning that the time-averaged decay curves  decays with the radiative rate *k*_tot_ = *k*_r_.^28,29^ Using the extracted radiative and nonradiative decay rates, we find that the emission efficiency is lowered to *η*_X*_ = 7.5% by trapping and subsequent nonradiative recombination of the charge-separated exciton to the ground state. From here on, we will label any excited state with a trap-related recombination pathway with an asterisk. For QD B, the amplitude in the OFF state increases with respect to the ON state (Figure 1g). This must be due to a boost of the radiative decay rate compared to the ON state. We quantify the relative radiative decay rate of the OFF state from the amplitude ratio and find a radiative decay rate enhancement of *k*_r,OFF_/*k*_r,ON_ = 1.88. This value of approximately 2 is consistent with intermittent charging/discharging (Figure 1h), where the quenched state is a trion (exciton with an additional delocalized charge carrier). In the trion state, the additional charge carrier doubles the number of radiative pathways.^24^ The emission efficiency is lowered by nonradiative Auger recombination in competition with radiative decay. Here, we assume that the additional delocalized charge carrier is an electron, because previous studies have identified that CdSe-based QDs tend to show negative-trion rather than positive-trion emission.^30,31^ We find a trion efficiency *η*_X^–^_ = 12.9%, which is indeed consistent with a negative trion, while positive trions should have lower efficiencies.^20^ For both QDs, we disentangled the radiative decay rates (green) from trap-assisted decay rate (blue, QD A) and the Auger decay rate (red, QD B) in Figure 1d,h.

In the regime of weak optical excitation (average number of excitons generated per pulse *n* ≪ 1), pulsed experiments allow us to distinguish between biexciton and exciton photons. We split the emission of a single QD using a conventional Hanbury-Brown–Twiss setup (Figure 2a), of which both detection channels employ an avalanche photodiode single-photon detector. We start by analyzing the biexciton in the ON state of QD B (Figure 1e for selected intensity range). The properties of the biexciton in the ON state of QD A are qualitatively similar and presented in the Supporting Information (Extended Data Section S6 for 5 single-QD measurements per quenching mechanism) The intensity-correlation function *g*^(2)^ (Figure 2b; histogram of photon-pair delay times) measured in the limit of weak optical excitation (*n* ≈ 0.15, see Supporting Information Figure S1), reveals the biexciton-to-exciton efficiency ratio η_BX_/η_X_ from the amplitude of the zero-delay peak *A*_0_ and the average amplitude of the side peaks *A*_±1_ = (*A*_–1_ + *A*_+1_)/2:^19^
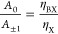
2By fitting a set of double-sided exponentials with amplitudes *A*_0_/*A*_±1_ and a flat background *B*, we find a biexciton efficiency of η_BX_ = 7.2% (assuming *η*_X_ = 100%), which is a typical value for CdSe/CdS/ZnS core–shell QDs.^19,32^ Note that both the zero-delay peak and the side peaks decay with the total exciton decay rate (Figure 2b, black fitted line) indicating that the stop photons in this experiment are mostly exciton emission events.

**Figure 2:**
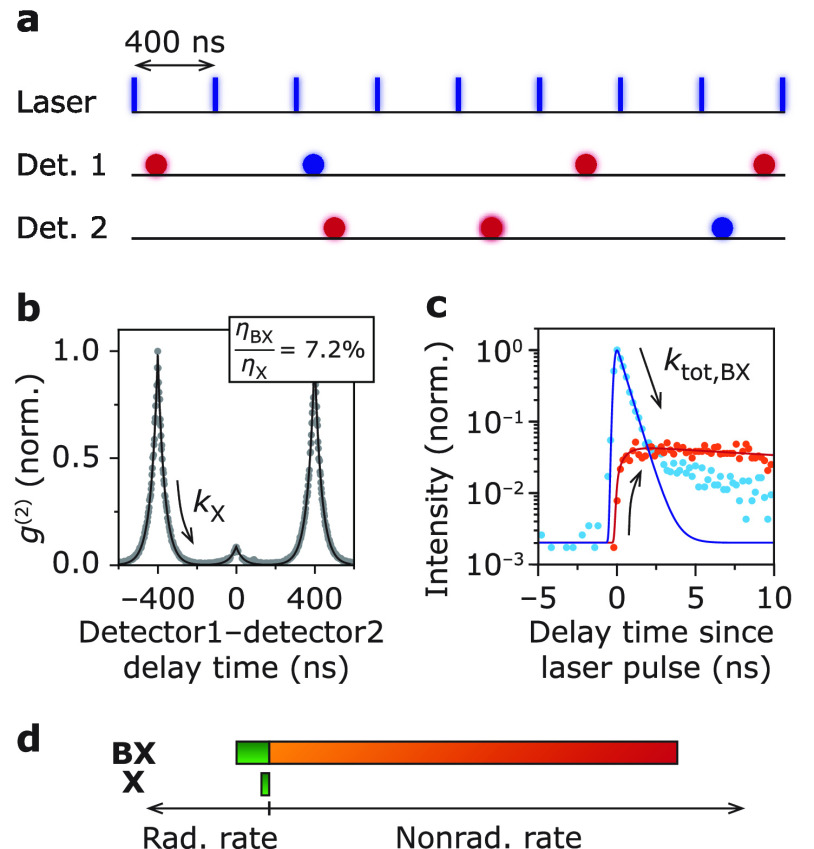
(a) Schematic depiction of a single-QD photon stream upon pulsed excitation. Every 400 ns, a laser pulse potentially brings the QD to the excited state, which potentially results in a photon-detection event on one of two single-photon detectors. Sometimes, the QD absorbs two quanta of energy driving the QD to the biexciton state, potentially leading to two photon-detection events after one laser pulse (blue–red photon pairs in photon stream). Note that in a real experiment the photon detections are sparser because of the low excitation fluences used. (b) Photon-correlation function of single QD B (Figure 1e–g). From the amplitude ratio between the zero-delay peak and the side peaks we determine the biexciton efficiency η_BX_ = 7.2%. (c) Cascaded decay curves of the first (blue) and second (red) photon of the biexciton cascade for QD B. The decay of the biexciton emission and the rise of the exciton emission go with the total biexciton decay rate *k*_tot,BX_. As expected, the cascaded decay curve of exciton emission decays with the total exciton decay rate *k*_tot,X_. (d) Radiative and nonradiative decay rates of the biexciton and exciton from single QD B (to scale), obtained by using the intensity-correlation measurements (Figure 2b,c) and time-averaged decay curves (Figure 1g).

We directly measure the excited-state dynamics of the biexciton cascade by constructing cascaded decay curves from the single-QD photon stream (Supporting Information S3). In this procedure, we select all photon-pair events (Figure 2a, blue–red photon pairs) and construct a decay curve of the first (blue dots, biexciton-to-exciton emission) and second (red dots, exciton-to-ground state emission) emission event of the biexciton cascade. In our experiments, cascaded decay curves typically contain only several tens to hundreds of photon-detection events, even over a relatively long single-QD measurement because of the low excitation fluence. Increasing the excitation fluence would increase the intensity of cascaded biexciton emission. However, this also corrupts the measurement by introducing emission events from higher multiexciton states. We use a maximum-likelihood estimation fitting procedure to extract reliable decay rates from these noisy decay curves (see Supporting Information Section S3.2 for details).^33^ From the biexciton-to-exciton decay curve (Figure 2c, dark blue line), we find a total decay rate of the biexciton *k*_tot,BX_ = 1.86 ns^–1^ for QD B (*k*_tot,BX_ = 2.75 ns^–1^ for QD A, Supporting Information Section S6). The slow component in the decay curve is attributed to exciton–background photon pairs and is neglected in the fitting procedure. The exciton-to-ground state decay curve (dark red line) shows a rise with *k*_tot,BX_ and decay with *k*_tot,X_, consistent with cascaded emission from the biexciton state to the ground state with average decay time ⟨*t*⟩ = *k*_tot,BX_^–1^ + *k*_tot,X_^–1^ since the laser pulse.

By combining the emission efficiency and total decay rate of the biexciton, we can disentangle the radiative and nonradiative recombination pathways (Figure 2d).^34^ We compute the relative radiative rate of the biexciton from
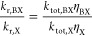
3which gives *k*_r,BX_/*k*_r,X_ = 4.2 for QD B (*k*_r,BX_/*k*_r,X_ = 3.9 for QD A), showcasing a 4-fold increase in the number of radiative pathways, consistent with statistical scaling.^25,34^ The increase of the radiative decay rate compared to the exciton can be explained by twice the number of charge carriers in the biexciton (2 holes and 2 electrons) compared to the exciton (1 hole and 1 electron).

To quantify the effect of opening/closing of a charge-carrier trap (QD A) and charging/discharging (QD B) on the biexciton efficiency, we construct the photon-correlation function *g*^(2)^ of the OFF state (Figure 3a,b; selected intensity range in Figure 1a,d). The quenched-biexciton-to-exciton efficiency ratio η_BX*_/η_X*_ in the OFF states follows from integrating the photon pairs in the side peaks and zero-delay peak. After subtracting different sources of background (see Supporting Information S3.2 for details) we find a quenched-biexciton-to-exciton efficiency ratio of η_BX*_/η_X*_ = (20 ± 3)% (Figure 3a) and a charged-biexciton-to-trion efficiency ratio of η_BX^–^_/η_X^–^_ = (27 ± 3)% (Figure 3b). Uncertainties are estimated by propagating Poisson noise and are quoted as one standard error. We convert these efficiency ratios to efficiencies of the quenched biexciton η_BX*_ = 1.5% and charged biexciton η_BX^–^_ = 3.5% using the trion and quenched-exciton efficiencies found in Figure 1c,g. Again, we disentangle the radiative and nonradiative decay rates (Figure 3c,d). Following the procedure introduced in eq 3, we compute the relative radiative decay rate of the charged biexciton compared to the neutral exciton *k*_r,BX^–^_/*k*_r,X_ = 3.2 and find roughly a 4-fold increase (average relative radiative decay rate 3.1 ± 0.1; mean ± standard deviation over 4 single-QD measurements). This indicates that the additional 1P electron in the charged biexciton (QD B) compared to the neutral biexciton does not directly participate in radiative recombination as was previously found by Shulenberger et al. for the triexciton state in CdSe/CdS core–shell QDs.^15^ The decay of the quenched biexciton BX* in QD A is so fast compared to the instrument response that it is difficult to quantify the radiative decay rate in this way. For our further analysis, we assume that it is equal to the radiative decay rate of the ON-state biexciton BX as we experimentally demonstrated for the exciton in the ON and OFF state in Figure 1c.

**Figure 3:**
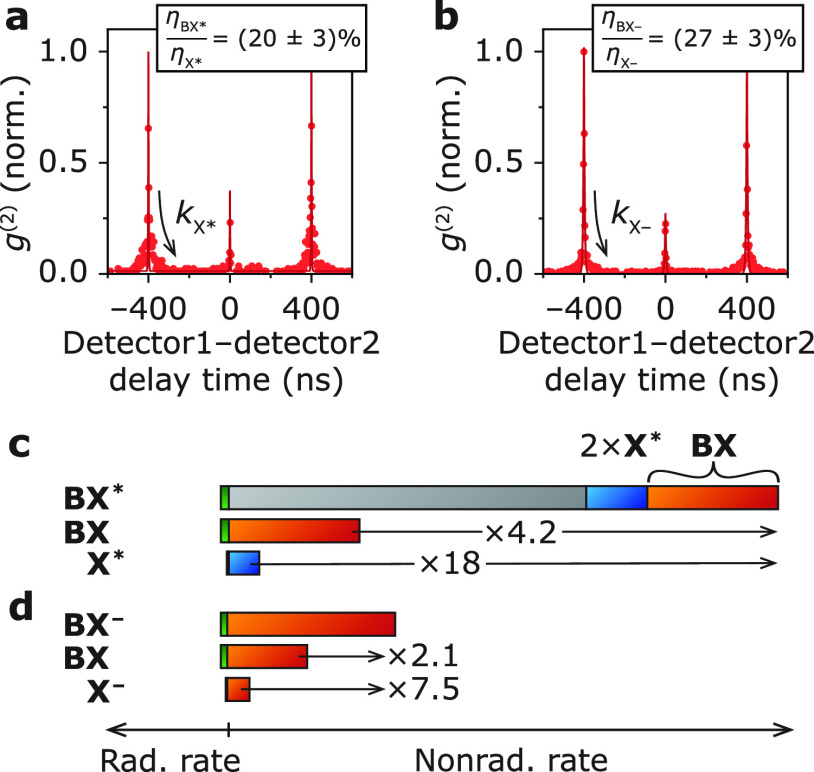
(a) Photon-correlation function *g*^(2)^ of the OFF state (QD A). From numerical integration of the peaks, we find a quenched-biexciton-to-exciton efficiency ratio η_BX*_/η_X*_ = (20 ± 3)%. The peaks decay with the quenched-exciton decay rate *k*_tot,X*_ = 0.62 ns^–1^. The fit (red solid line) disregards the slow component due to mixing in the ON state. (b) Same as panel a, but for QD B, where an additional delocalized charge carrier quenches the luminescence. The peaks decay with the quenched-exciton decay rate *k*_tot,X^–^_ = 0.46 ns^–1^. We find a charged-biexciton-to-trion efficiency ratio η_BX^–^_/η_X^–^_ = (27 ± 3)%. (c) Disentangled radiative and nonradiative decay rates of the quenched biexciton, biexciton, and quenched exciton to scale. The nonradiative decay rate of the quenched biexciton state BX* is boosted by a factor of 4.2 ± 1.2 compared to the regular biexciton and a factor of 18.0 ± 2.1 compared to the quenched exciton. From statistical scaling, we can at least expect the nonradiative decay rate of the quenched biexciton to contain the Auger recombination rate of the biexciton plus twice the electron-trapping rate of the quenched exciton. To explain the excess of the nonradiative decay rate (gray) in the quenched biexciton, the electron-trapping rate must be boosted a factor of 14.7 ± 2.6 compared to the quenched exciton. (d) Same as panel c, but for QD B. The nonradiative rate of the charged biexciton increases with a factor of 2.1 ± 0.3 compared to the neutral biexciton and a factor of 7.5 ± 0.5 compared to the trion.

We quantify the nonradiative decay rate of the biexciton (QD A and B), quenched biexciton BX* (QD A) and the charged biexciton BX^–^ (QD B) using
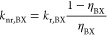
4and find *k*_nr,BX*_ = 11.2 ns^–1^ (QD A; compared to *k*_nr,BX_ = 2.68 ns^–1^) and *k*_nr,BX^–^_ = 3.42 ns^–1^ (QD B; compared to *k*_nr,BX_ = 1.63 ns^–1^). Figure 3c,d shows the radiative and nonradiative decay rates of exciton (OFF state) and biexciton (ON and OFF state) of QD A and B to scale. We find that the nonradiative decay rate of the charged biexciton BX^–^ is boosted by a factor of 2.1 ± 0.3 compared to the biexciton BX and by a factor of 7.5 ± 0.5 compared to the trion X^–^, consistent with ensemble-scale measurements on CdSe/ZnS core–shell QDs and single-dot measurements performed under strong optical excitation.^35,36^ On average the nonradiative decay rate of the charged biexciton is boosted by a factor of 2.0 ± 0.2 compared to the neutral biexciton (mean ± standard deviation of 5 QDs from the same synthesis batch). Apparently, the presence of a delocalized 1P electron increases the Auger recombination rate in the charged biexciton BX^–^ compared to the neutral biexciton BX. For QD A, where the emission in the OFF state is quenched by open charge-carrier traps, we use the same procedure and observe that the nonradiative decay rate of the quenched biexciton BX* is boosted by a factor of 4.2 ± 1.2 compared to the biexciton BX and by a factor of 18.0 ± 2.1 compared to the quenched exciton X* (Figure 3d). On average the nonradiative decay rate of the quenched biexciton is boosted by a factor of 3.9 ± 1.2 compared to the biexciton (mean ± standard deviation of 5 QDs from the same synthesis batch). These boost factors exceed the values found previously in single-QD measurements under strong optical excitation.^36^ We hypothesize that the strong optical excitation of the previous experiments^36^ hindered the characterization of the biexciton efficiency in the OFF-state because of interference from other (charged) (multi-) exciton emissions and flickering.^24,37,38^

In the conventional picture of trap-assisted recombination, nonradiative decay of the quenched exciton X* in the OFF state is a two-step process where (1) the electron gets trapped, after which (2) the localized electron and delocalized hole recombine nonradiatively.^8^ Here, the quantum yield η_X*_ = *k*_r_/(*k*_r_ + *k*_t_) is determined solely by the trapping rate *k*_t_ which competes with radiative recombination *k*_r_, but not on the nonradiative recombination rate *k*_nr_ from the charge-separated state. We can assume that the quenched biexciton BX* has access to at least the Auger pathway of the biexciton and the trap-assisted pathway of the quenched exciton X*. From statistical scaling, we expect that the Auger rate of the quenched biexciton is equal to that of the biexciton, while the rate of charge-carrier trapping may be doubled for BX* compared to X*. However, in Figure 3d we observe that the nonradiative decay rate of the quenched biexciton exceeds the sum of the BX Auger (red) and the X* trap-assisted recombination rate (blue). A significant part of the nonradiative decay rate is unaccounted for (gray). The BX* electron trapping rate would need to be higher than in the X* state by a boost factor of *K* = (*k*_nr,BX*_ – *k*_nr,BX_)/*k*_nr,X*_ = 14.7 ± 2.6 within the conventional picture of trap-assisted recombination. This far exceeds the predicted boost from statistical scaling.

A possible explanation for the discrepancy between the simplest picture of trap-assisted recombination and the experiment might be that the electron wave functions reorganize in the biexciton state due to electrostatic interactions, increasing the wave function overlap with trap states compared to the exciton. To first order, we expect the electron-trapping rate to scale with the overlap between the delocalized electron density (wave function squared) and the trap.^39,40^ To test the effect of reorganizing wave functions, we set up a simple quantum-mechanical effective-mass model of multicarrier states in a core–shell QD acting as a potential well for holes (confined to the CdSe core) and electrons (confined to the QD). Coulomb interactions between carriers are included, and the reorganized wave functions are constructed from a basis set of particle-in-a-spherical-box states (section S4 in the Supporting Information). We consider two QD geometries: (1) a concentric core–shell QD and (2) a core–shell QD with an off-centered core. In both cases, we observe that the enhanced electrostatic attractions in the biexciton state compared to the exciton state increase the electron density near the CdSe core, possibly explaining the extracted boost factor *K*. However, we find that the electron density in the biexciton is at most boosted by only a factor of 1.3 compared to the exciton at any location in the QD. Therefore, we conclude that—whatever the actual position of the trap state—electrostatic interactions cannot explain the boost of the apparent electron-trapping rate in the biexciton.

Because wave function distortions cannot reproduce the boosted electron-trapping rate measured in our experiments, we need an alternative model for trap-assisted recombination in the OFF state. We propose that during trap-induced OFF periods, the QD switches rapidly between a state with delocalized charge carriers (X′ or BX′, Figure 4) and a charge-separated state with one trapped charge carrier and the other charge carriers delocalized (X″ or BX″). In both the delocalized and the charge-separated states the QD has an accessible trap state. Filling of the trap state by a charge carrier (X″) introduces nonradiative trap recombination. State X′ can decay nonradiatively only by going via state X″. Very fast trapping *k*_t_ and detrapping *k*_dt_ compared to excited-state decay leads to quasi-steady-state populations of the delocalized and charge-separated states (details in Supporting Information Section S5). As a consequence, the quenched exciton X* and biexciton BX* decay radiatively or nonradiatively through effective decay rates that are population-weighted averages of those of the delocalized states and charge-separated states. A small quasi-steady-state population of the charge-separated state X″ (or BX″) can already strongly quench the luminescence, if the nonradiative decay rate of this state is sufficiently fast.

**Figure 4:**
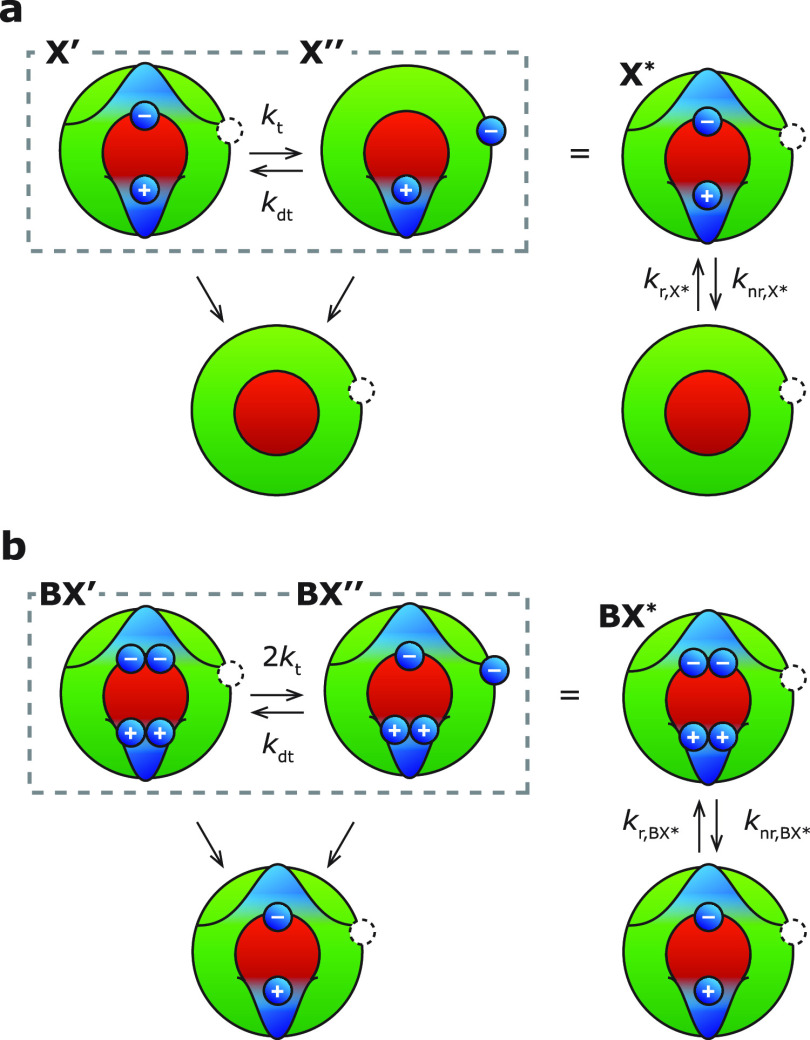
Proposed model for trap-assisted recombination of exciton and biexciton. (a) In the quenched exciton X*, the QD has an accessible trap state and switches rapidly between a delocalized exciton state X′ (empty trap state) and a charge-separated state X″ (filled trap state). The quenched exciton has effective radiative and nonradiative decay rates, which are the population-weighted averages of the exciton state with delocalized carriers X′ and the charge-separated exciton state X″. (b) Same as panel a, but for the quenched biexciton BX*. We assume that the electron-trapping rate is doubled with respect to the quenched exciton due to statistical scaling. The quenched biexciton BX* rapidly switches between a biexciton state with delocalized carriers BX′ and a trion with a filled electron trap BX″. The localized charge carrier is a very efficient Auger acceptor, decreasing the efficiency of the quenched biexciton BX*.

Our revised model for trap-assisted recombination matches the experiments for very reasonable values of the rate constants. In our experiments, we extract equal radiative decay rates of the exciton in the ON and OFF state from the time-averaged decay curves (*A*_OFF_/*A*_ON_ ≈ 1, Figure 1c and QDs 5–10 in the Supporting Information Section S6). In the quenched exciton state X*, the QD switches between a bright delocalized exciton X′ and a nonemissive charge-separated exciton X″ (Figure 4a). Our model reproduces an effective radiative decay rate of the quenched exciton X* equal to that of the exciton X in the ON state, if the quasi-steady-state population of the delocalized exciton X′ is almost unity. The electron detrapping rate *k*_dt_ must therefore be much faster than the trapping rate *k*_t_. This implies that the trapping energy Δ*E* = *k*_B_*T* ln(*k*_dt_/*k*_t_), with *k*_B_*T* the thermal energy, is positive; that is, the trap state involved in quenching lies outside the band gap. Some QDs from the same synthesis batch (and reports from literature on CdSe/CdS dot-in-rods^31^ and CdSe/CdS core–shell QDs^41^) showed lower radiative decay rate of the OFF state *A*_OFF_/*A*_ON_ < 1, indicating that the quasi-steady-state populations of regular and charge-separated exciton states are more comparable (Supporting Information Figure S8) and the trap state is approximately resonant with the band edge. For small quasi-steady-state population of the charge-separated exciton X″, its nonradiative decay rate needs to be much higher than the radiative decay rate of the delocalized exciton X′ to explain the low efficiency of the quenched exciton X* (η_X*_ = 7.5%). The nonradiative decay pathway of the charge-separated exciton X″ is illustrated in the configuration-coordinate diagram of Figure 5a, which depicts the energies of delocalized and charge-separated exciton states along with the geometrical distortions due to charge carrier localization. The charge-separated state X″ can decay nonradiatively by crossover to the ground state (green arrow). This is, effectively, recombination of the localized charge carrier with the remaining delocalized charge carrier. In contrast, the delocalized exciton state cannot undergo this crossover process, because geometrical distortions in the delocalized exciton X′ compared to the ground state are negligible if charge carriers remain delocalized. Geometrical reorganization upon charge-carrier trapping has been invoked before by Mooney et al. for CdSe-based QDs to explain red-shifted and broadband trap emission from QDs without the involvement of midgap trap states.^42^ Quantitative understanding of the transitions between delocalized and charge-separated states probably involves considerations beyond classical Marcus theory.^42−44^ Temperature-dependent single-QD measurements in combination with density-functional theory calculations might be necessary to further test our proposed nonradiative recombination model and understand the nature of the trap state involved.

**Figure 5:**
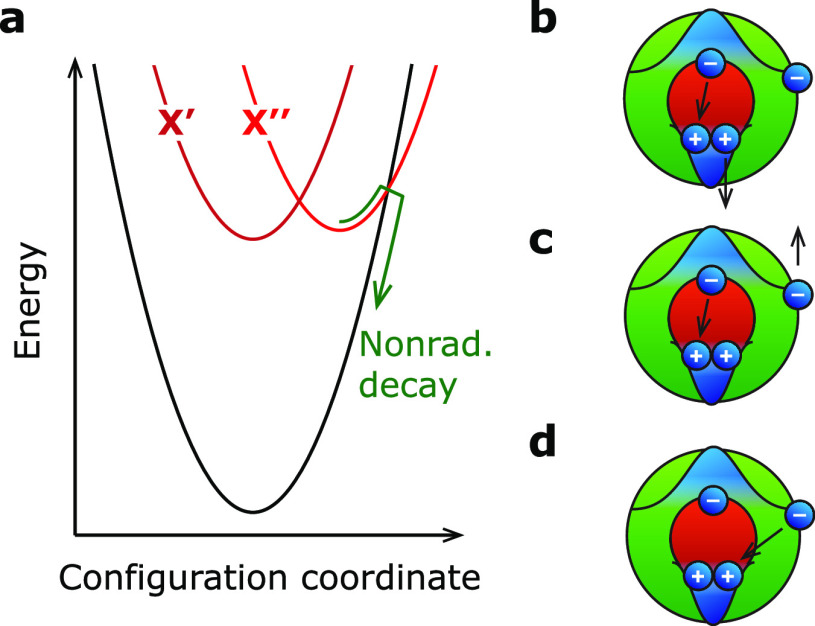
(a) Configuration-coordinate diagram of the ground state (black), delocalized exciton X′ (dark red), and charge-separated exciton X″ (light red). For the charge-separated state X″, nonradiative trap recombination can take place directly by (thermally activated) crossover to the electronic ground state. (b–d) Nonradiative recombination mechanisms of the charge-separated biexciton BX″. The biexciton can decay nonradiatively via (b) the positive-trion pathway, (c) trap-assisted Auger recombination, and (d) direct trap recombination. Figure S8 in the Supporting Information shows how the quenching pathways of the charge-separated biexciton BX″ can be visualized in a configuration-coordinate diagram.

The observed efficiency of the quenched biexciton BX* η_BX*_ = 1.5% is reproduced by the model, using an intraband trap level (*k*_dt_/*k*_t_ ≫ 1), if we plug in a nonradiative decay rate of the charge-separated biexciton BX″ of *k*_nr,BX″_/*k*_nr,X″_ = *K*/2 ≈ 7 (see Supporting Information Section S5 for derivation). This rapid nonradiative recombination rate of the charge-separated biexciton BX″ is understandable by considering the available nonradiative recombination pathways of the charge-separated biexciton BX″ (Figure 5b–d): the efficient positive-trion pathway (Figure 5b, top),^20^ trap-assisted Auger recombination (Figure 5c), and direct trap recombination (Figure 5d). Trap-assisted Auger recombination can be particularly fast because of alleviation of the momentum-conservation selection rule,^45,46^ as has been observed before for ZnO and CuInS_2_ nanocrystals.^47,48^ The trap-assisted Auger pathway (Figure 5c) and direct trap recombination (Figure 5d) are not active for the ON-state biexciton, hence the much faster nonradiative decay of the charge-separated biexciton BX″ and the much lower overall efficiency of the biexciton BX* in the trap-induced OFF state. Trap-assisted recombination is also unavailable for the trion state X^–^ observed in QD B (Figure 1e–h). The trion state X^–^ might have an ejected localized charge carrier on or near the QD surface, but because the charged OFF state of QD B persists over many optical cycles (OFF periods of up to 100 ms, Figure 1e) we know that the ejected charge carrier is inactive and does not contribute to recombination.

The revised model for trap-assisted recombination (QD A) and fast Auger recombination of charged (bi)excitons (QD B) has potential implications for lasing using colloidal QDs. One could expect a considerable fraction of QDs in a gain medium to be charged or to have an open trap state at high excitation fluences, as was shown before by power-dependent blinking studies.^8^ Hence, while several groups purposely charged a gain medium to benefit pulsed lasing,^49,50^ charging also happens spontaneously due to blinking. In practice, a gain medium probably contains a distribution of charged QDs, QDs with open electron traps, and QDs in the ON state. The effect of blinking, by charging and opening of electron traps, on *pulsed lasing* is two-sided. Reaching population inversion in QDs with open electron traps is more difficult because the traps act as a storage site for electrons, effectively decreasing the population inversion of band-edge levels (Figure 6a).^49,51^ To quantify the effect of electron storage on the pulsed-lasing performance, we compute a gain threshold (where net absorption is zero) in terms of excitons generated per pulse *n*_th_ in the presence of open electron traps (Figure 6c). The exact gain threshold depends on the quasi-steady-state population of the delocalized (DL) and charge-separated (CS) states. In the absence of trapping *k*_t_/*k*_dt_ ≪1, the population of the delocalized state is unity. In this case, we find a gain threshold of *n*_DL,th_ = 1.15 as was found before by Kozlov et al. for QDs in the ON state.^49^ On the other hand, if trapping is much faster than detrapping *k*_t_/*k*_dt_ ≫ 1 the QD mainly populates the charge-separated state and the gain threshold increases to *n*_CS,th_ = 1.92. On the other hand, charging has a positive effect on the lasing performance since the excess electron bleaches absorption and increases the stimulated emission pathways (Figure 6d). This lowers the lasing threshold to *n*_C,th_ = 0.58 for a gain medium with QDs containing exactly one excess electron (Figure 6e, red). In a practical gain medium, the distribution of charged QDs, QDs with open traps, and QDs in the ON state determines the overall gain threshold. Achieving *continuous-wave lasing* from charged QDs and QDs with an open trap state is complicated. Trap-assisted recombination introduces very fast nonradiative decay, which makes achieving a high steady-state exciton or biexciton occupation difficult. Even charging—which is beneficial for pulsed lasing—may be undesirable since both the trion and the charged biexciton have a reduced gain lifetime compared to the neutral exciton states.^52^

**Figure 6:**
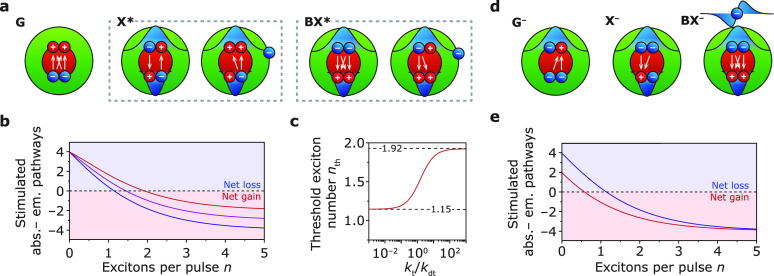
(a) Schematic of ground state G, quenched exciton X*, and quenched biexciton BX* including all stimulated emission (downward arrows) and absorption (upward arrows) pathways. (b) Net absorption in terms of the difference between absorption and stimulated emission pathways as a function of average number of excitons generated per laser pulse *n* (assuming Poisson statistics) for *k*_t_/*k*_dt_ ≪1 (blue), *k*_t_/*k*_dt_ = 1 (purple), and *k*_t_/*k*_dt_ ≫ 1 (red). We assume that the steady-state populations of charge-separated and delocalized states are established on time scales faster than stimulated emission and that multiexcitons higher than biexcitons do not contribute to the emission and/or filling of the trap state. We observe that the gain threshold in terms of excitons generated per pulse *n*_th_ (where net absorption is zero) is higher for high population in the charge-separated exciton states because the localization of the charge carrier decreases population inversion of the band-edge levels. (c) Threshold exciton number *n*_th_ as a function of the trapping/detrapping ratio. In the limit of small trapping/detrapping ratio *k*_t_/*k*_dt_ ≪1, QDs populate the delocalized exciton states. This leads to a lasing threshold of 1.15 excitons generated per pulse for a collection such QDs in a gain medium.^49^ In the limit of large trapping/detrapping ratio *k*_t_/*k*_dt_ ≫ 1, QDs populate the charge-separated exciton states. A gain medium with such QDs has significantly higher lasing threshold of 1.92 excitons generated per pulse. (d) Same as panel a, but for a charged ground state G^–^, exciton X^–^, and biexciton BX^–^. (e) Same as panel b, but for the neutral exciton states (blue) and for the charged exciton states (red). Here, the gain threshold number *n*_th_ decreases from 1.15 in the regular exciton states to 0.58 in the charged exciton states. The subunity threshold for lasing has been shown experimentally for charged CdSe-based QDs.^49^

To summarize, we have measured “blinking” of the biexciton efficiency during charging/discharging and opening/closing of charge-carrier traps using intensity-gated photon correlation analysis. For a complete overview, the extended data in Supporting Information Section S6 contains all extracted excited-state decay rates and emission efficiencies of the single-QD measurements in this work. This work contains results on a relatively low number of single QDs because the measurement is very time-consuming. The requirement of weak optical excitation combined with the nonlinear dependence of photon-pair counts on emission efficiency makes measurement time of approximately 20 min necessary for sufficient biexciton photon pairs in the OFF state. Additionally, many single-QD measurements are unusable due to flickering,^24,37,38^ making it difficult to isolate a particular emissive state which makes a fair comparison of the extracted rate constants impossible. To obtain clean data with sufficient counts, we thus had to focus on the brightest QDs with clearly resolvable emissive states in the measurement series.

We found that the additional 1P electron in the charged biexciton doubles (2.0 ± 0.2; mean ± standard deviation averaged over 5 single QDs) the nonradiative decay rate compared to the neutral biexciton. Open charge-carrier traps quadruple (3.9 ± 1.2; mean ± standard deviation averaged over 5 single QDs) the nonradiative decay rate of the quenched biexciton BX* compared to the biexciton BX. The dramatic increase of the nonradiative rate for the biexciton with open charge-carrier traps pointed toward a revisited model for trap-assisted recombination with reversible electron trapping. Although the variations of the nonradiative decay rate boost in the quenched biexciton BX* are large, all extracted boost factors are significantly larger than expected based on the conventional model for trap-assisted recombination. Future work, for example density-functional theory or spectroscopy on the temperature dependence of blinking, could test our proposal of nonradiative recombination via a reversible trap level higher in energy than the band edge. Alternatively, extension of recombination models based on multiple trap levels^26,53^ inside the bandgap to biexciton recombination might offer an explanation for our experimental data.

## Supporting Information Available

Experimental methods, details of data processing, description of quantum-mechanical calculations, description of the trap-assisted recombination with reversible trapping, and overview of all single-QD experiments (PDF)
