# Supplementary material for: Biexciton Blinking in CdSe-Based Quantum Dots
Source: J Phys Chem Lett. 2023 Jun 5;14(23):5353–61. doi: 10.1021/acs.jpclett.3c00437 (PMC10278138; doi:10.1021/acs.jpclett.3c00437)
Supplement: Supplementary file 1 — jz3c00437_si_001.pdf [file jz3c00437_si_001.pdf]

Supporting Information for

Biexciton Blinking in CdSe-based Quantum Dots

Sander J.W. Vonk [1], *and Freddy T. Rabouw* [1*]

[1]Debye Institute, Utrecht University, Princetonplein 1, 3584 CC Utrecht, The Netherlands

[*] Corresponding Author: f.t.rabouw@uu.nl

S1 Synthesis and characterization of core–shell quantum dots

S1.1 CdSe core synthesis

The synthesis of the CdSe cores was based on a method by Chen et al.[S1] Octadecylphosphonic acid (ODPA, 280 mg), trioctylphosphine oxide (TOPO, 3 g), and cadmium oxide (CdO, 60 mg) were added to a three-neck flask (50 mL) and degassed for one hour at 150°C. The temperature was raised to 320°C and trioctylphosphine (TOP, 1 mL) was added. The mixture was heated to 380°C, and a selenium precursor (Se 60 mg in 0.5 mL TOP) was injected to initiate the core growth. Core growth was allowed to continue for approximately 25 s. The synthesized cores were washed with methyl acetate (1:1 volume ratio), centrifuged, and redispersed in hexane. The product was filtered (Millipore filters 0.2 µm) and the washing steps described above were repeated once. The sample was stored in a glovebox.

S1.2 CdS and ZnS shell growth

To produce the Cd-oleate precursor, Cd-(acetate)$_2$ (1.32 g) was dissolved in octadecene (ODE, 52.4 g) and oleic acid (OA, 7.4 g) and heated to 120°C for three hours. After three hours, the Cd-oleate solution was cooled down and stored in a glovebox. Zn-oleate was made in a similar manner by adding together zinc acetate (0.16 g), OA (1 g), ODE (1.6 mL) and oleylamine (OLAM, 1.6 mL). The mixture was heated to 130°C and afterwards stored in a glovebox.

The growth of the CdS and ZnS shells is based on previous methods from Chen *et al.*, Boldt *et al.* and Hanafi *et al.*[S1–S3] First, the CdS shell was grown. The CdSe cores (50 nmol) and ODE (3 mL) were added to a 100 mL three-neck flask and subsequently degassed. The temperature was raised to 310°C. At 240°C, Cd-oleate and 1-octanethiol (0.075 M in ODE) were injected slowly to grow a CdS shell at a rate of 1 monolayer per hour until a final thickness of 8 monolayers. The CdSe/CdS QDs were degassed at 120°C before growing the final ZnS shells. To grow the ZnS shells, the QD solution was heated to 280°C. At 210°C, Zn-oleate and 1-octanethiol (0.55 M in ODE) were injected to grow a total of 2 monolayers of ZnS shell at a rate of 1 monolayer ZnS per hour. The product was washed twice with a methanol:butanol mixture (1:2), centrifuged, washed again with methyl acetate, which was followed by another centrifugation step. Finally, the QD solution was stored in a glovebox.

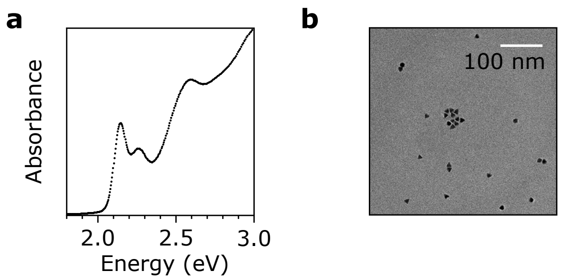

Figure S1 | Characterization of quantum dot sample. (a) Absorption spectrum of the CdSe cores used as seeds for the CdS/ZnS shell growth. From the spectral position of the 1S absorption (577 nm), we obtain an average core size of 1.93 nm using a sizing curve.[S4] (b) Transmission electron micrograph of the CdSe/CdS/ZnS core–shell–shell sample used in all experiments in this Letter. The electron microscopy images were acquired with a JEOL JEM-1400 plus transmission electron microscope.

S2 Experimental setup

All single-QD measurements and ensemble time-resolved emission measurements were performed on a home-built optical setup consisting of a Nikon Ti-U inverted microscope body. The single-QD samples were prepared by spincoating a diluted (10^4 dilution factor) solution of QDs on a glass coverslip, which was then placed on the microscope. A 405-nm pulsed laser (Picoquant D-C 405, controlled by Picoquant PDL 800-D laser driver) was guided to the sample by a dichroic mirror (edge at 425 nm, Thorlabs DMLP425R) and focused by an oil-immersion objective (Nikon CFI Plan Apochromat Lambda 100×, NA 1.45) onto the sample. The QD emission was collected by the same objective and guided to two single-photon detectors in a Hanbury Brown–Twiss setup. The emission was split by a non-polarizing beamsplitter (Thorlabs BS013) and focused (achromatic aspherical lens Edmund optics, 49-659) onto one of two avalanche photodiodes (APD; Micro Photonic Devices PDM, low dark counts <5 Hz).
Both APDs and the laser driver were connected to a quTools quTAG time-to-digital converter, which communicated all photon detection events and laser pulses to a computer. Home-written software was used for live data visualization (*e.g.* photon-correlation function, decay curve, and intensity trace) and data storage.[S5]

For the single-QD measurements, we estimated the average excitons generated per pulse n from a saturation measurement on a single QD (Figure S1). Figure S1a shows the single-exciton emission intensity as a function of excitation fluence (10 s time interval per excitation fluence), where multiexciton emission is discarded using a gating procedure (delay times $t \geq 2$ ns). We estimate the single-exciton emission intensity in the ON state as a function of excitation fluence from the mean counts of the 20 most intense bins per measurement interval (Figure S1b, blue dots). In theory, the single-exciton emission intensity I_X is given by

$$I_\mathrm{X} = AP_{\geq 1} = A(1 - \mathrm{e}^{-n}) \tag{1}$$

where $P_{\geq 1}$ is the probability to form at least 1 exciton per laser pulse, and A is proportional to the repetition rate, the binning time, and the collection efficiency of our microscope setup. The exciton formation probability $P_{\geq 1}$ depends on the excitation fluence J via $n = \sigma CJ/\hbar\omega$, where σ is the absorption cross-section, $\hbar\omega$ the photon energy, and C an arbitrary scaling factor to account for the fact that we only know the relative excitation fluences. We fit eq 1 to the blue data points with fit parameters A and σC. Based on the fitted prefactor A, we compute a collection efficiency of 8.5% for our microscope setup. Using the fitted value for σC, we can convert the excitation fluence J axis to an excitons generated per pulse n axis (Figure S1b). We estimate an average excitons per pulse n between 0.1–0.2 in our experiments. Using these excitation fluences, we are able to isolate the cascaded emission of the biexciton without significant contribution of higher multiexcitons (no more than 0.5 percentage points deviation of the measured biexciton efficiency compared to the real biexciton efficiency). We estimate that these

excitation fluences at most 0.5%. This procedure might also introduce a systematic error. Determining a reliable value for n heavily relies on quantifying the emission intensity of the ON state as a function of excitation fluence. However, at high excitation fluence (T = 80–100 s, blue square in Figure S1a,b) the QD switches very fast between the ON and OFF state, which makes it impossible to isolate ON-state emission using a binning procedure. Including these data points in the fit would introduce a systematic error on the determination of the excitons generated per pulse n.

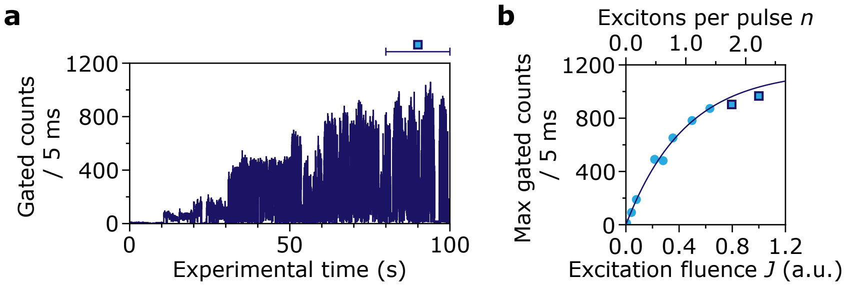

Figure S2 | Determining the excitons generated per pulse n from emission saturation. (a) Single-exciton emission intensity I_X as a function of excitation fluence (10 s time interval per fluence). **(b)** Mean counts of the 20 most intense bins as a function of excitation fluence (blue dots). By fitting eq 1 to the blue data points (blue solid line) we can convert the excitation fluence to a excitons per pulse n. We estimate an average excitons per pulse n between 0.1–0.2 in our experiments. Including the high-excitation data points (blue squares) would introduce a systematic error in the determination of the average excitons generated per pulse n, since rapid switching makes isolation of ON-state emission impossible using a binning procedure.

S3 Analysis procedures

S3.1 Quenching mechanisms of quenched excitons

To characterize the quenching mechanism of the exciton in our single-QD measurements, we need to quantify all radiative and nonradiative rates in the ON and OFF state. We select the ON ad OFF state using an intensity-gating (per 5 ms) procedure, where the exact intensity range and thresholds are selected manually by inspecting the intensity trace. The experimental decay curves are time-averaged by dividing by the number of 5-ms bins within the selected intensity range. For a certain emissive state, in general, a time-averaged decay curve $\overline{I(t)}$ is given by:

$$\overline{I(t)} \propto k_\mathrm{r} \mathrm{e}^{-k_\mathrm{tot} t}. \tag{2}$$

Here, the amplitude of the decay curve A is proportional to the radiative decay rate k_r, while the slope of the decay curve is equal to the total decay rate $k_\mathrm{tot} = k_\mathrm{r} + k_\mathrm{nr}$—containing all radiative and nonradiative processes. In our experiments, the laser fluence remains constant and we assume that the absorption-cross section also remains constant at the laser wavelength of 405 nm. Any change of the decay-curve amplitude in the OFF state compared to the ON state can be due to (1) a change in the radiative decay rate, (2) mixing between different excited states with different oscillator strengths (for example bright and dark fine-structure states), or (3) excited-state decay within the instrument response. We distinguish between two types of excited-state decay within the instrument response: rapid decay of band-edge excitons or electron/hole trapping from a hot-carrier state effectively lowering the number of band-edge excitons generated per laser pulse. Opening and closing of hot-carrier traps—labelled *B-type blinking* by Galland *et al.*—is not observed in our sample.[S6]

We will investigate the influence of the instrument response on a rapidly decaying emissive state. In our experiments, we measure the convolution of the excited-state decay with a Gaussian instrument response, given by:

$$\overline{I(t)} = \frac{A}{2} \mathrm{e}^{k_\mathrm{tot}^2 \sigma^2/4} \mathrm{e}^{-k_\mathrm{tot}(t-\Delta)} \left[1 + \mathrm{erf}\Big(\frac{t-\Delta}{\sigma} - \frac{k_\mathrm{tot}\sigma}{2}\Big)\right] + B \tag{3}$$

where Δ and σ are the offset and width (standard deviation) of the Gaussian instrument response, A is the decay-curve amplitude (proportional to the radiative decay rate) and B is the amplitude of a flat background signal. We characterized the instrument-response function of our experimental setup by measuring a histogram of photon-arrival times of the laser reflecting off a silver mirror mounted on our sample stage (Figure S2a). Such a histogram is broadened due to the pulse duration of the laser and the electronic processing of the photon-detection events by the APD and time-tag box. The width of the slightly asymmetric instrument response is obtained by fitting a two-sided Gaussian to the histogram (σ_- for $t < \Delta$ and σ_+ for $t > \Delta$) and we find $\sigma_- = 0.22$ ns and $\sigma_+ = 0.24$ ns. To illustrate the effect of the instrument response on the determination of the excited-state decay parameters, we plot theoretical decay curves with unity amplitude $A = 1$ and background $B = 10^{-3}$ for different total decay rates $k_{\text{tot}} = \{0.1, 1, 10\}$ ns^{-1} at a fixed symmetric instrument-response width $\sigma = 0.24$ ns. We observe that the apparent decay-curve amplitude decreases with increasing total decay rate due to excited-state decay within the instrument response. At the highest total decay rate, we observe purely the Gaussian instrument response and quantifying the input amplitude and decay rate is impossible. By decreasing the instrument-response width for the highest total decay rate (Figure S2c), we recover the input amplitude. When $k_{\text{tot}}\sigma \to 0$ we retrieve the single-exponentially decaying function

$$\lim_{k_{\text{tot}}\sigma \to 0} I(t) = A\mathrm{e}^{-k_{\text{tot}}(t-\Delta)} H(t-\Delta) + B \tag{4}$$

where $H(t-\Delta)$ is the Heaviside theta function. In the main text, we fit all experimental decay curves to eq 3 to account for the effect of a Gaussian instrument response and flat background on determining the decay-curve amplitude and the total decay rate.

In our experiments, we assume the ON state has unity efficiency, which has previously been proven by Brokmann *et al.* by varying the local density of optical states for single QDs.[S7] In that case, the QD decays to the ground state only via radiative processes and the total decay rate equals the radiative decay rate. We can determine the radiative decay rate $k_{\text{r,OFF}}$, nonradiative decay rate $k_{\text{nr,OFF}}$ and the efficiency η_{OFF} of the OFF state using:

$$k_{\text{r,ON}} = k_{\text{tot,ON}} \tag{5a}$$

$$k_{\text{r,OFF}} = \frac{A_{\text{OFF}}}{A_{\text{ON}}} k_{\text{r,ON}} \tag{5b}$$

$$k_{\text{nr,OFF}} = k_{\text{tot,OFF}} - k_{\text{r,OFF}} \tag{5c}$$

$$\eta_{\text{OFF}} = \frac{k_{\text{r,OFF}}}{k_{\text{tot,OFF}}}. \tag{5d}$$

Note that we determine the efficiency of the OFF state from the excited-state decay dynamics of the ON and OFF states. In principle, we could have also more directly measured the efficiency of the OFF state from the relative photon-emission rate visible in the intensity trace. However, the low photon emission rate makes this analysis dependent on a correct subtraction of the background photon-emission rate. We checked that both analysis procedures yield similar efficiencies of the OFF state. This strengthens our assumption that B-type blinking is absent in our sample. For B-type blinking, such an analysis procedure based on decay rates would yield unity efficiency of the OFF state, which is clearly inconsistent with the emission intensities of the ON and OFF states.

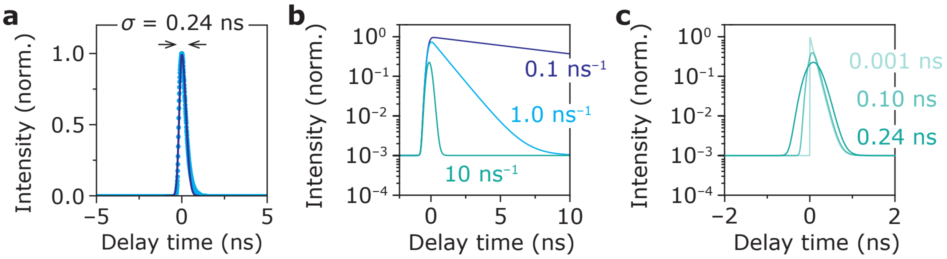

Figure S3 | Influence of instrument-response function on excited-state decay measurements. (a) Histogram of photon-arrival times of the 405-nm diode laser, measured in reflection off a silver mirror. We extract the width of the instrument-response function by fitting a two-sided Gaussian (σ_- for $t < \Delta$ and σ_+ for $t > \Delta$) and find $\sigma_- = 0.22$ ns and $\sigma_+ = 0.24$ ns. **(b)** Theoretical decay curves with varying total decay rates $k_{\mathrm{tot}} = \{0.1, 1, 10\}$ ns^{-1} for a fixed symmetric instrument-response width $\sigma = 0.24$ ns. The apparent decay-curve amplitude decreases for higher decay rates due to excited-state decay within the instrument response. **(c)** Theoretical decay curves for a total decay rate $k_{\mathrm{tot}} = 10$ ns^{-1} with varying width of the instrument response $\sigma = \{10^{-3}, 0.1, 0.24\}$ ns. By decreasing the width of the instrument response, we recover the input decay-curve amplitude $A = 1$.

S3.2 Quenching mechanism of (quenched) biexcitons

S3.2.1 Emission efficiency of (quenched) biexcitons

We characterize the efficiency and excited-state dynamics of the biexciton in the ON and OFF state from intensity-correlation analysis. In Figures 2 and 3 of the main text, we construct the intensity-gated intensity-correlation functions of both states. For the ON state, we determine the biexciton efficiency by fitting a set of double-sided exponentials using a least-squares fitting procedure

$$g^{(2)}_{\mathrm{ON}}(\tau) = A_{\pm 1}\left(\mathrm{e}^{-k_{\mathrm{tot,X}}|\tau - T|} + \mathrm{e}^{-k_{\mathrm{tot,X}}|\tau + T|}\right) + A_0\mathrm{e}^{-k_{\mathrm{tot,X}}|\tau|} + B, \tag{6}$$

where A_0 is the amplitude of the zero-delay peak, $A_{\pm 1}$ the amplitude of the side peaks, B the amplitude of the flat background, $T = 400$ ns is the time between laser pulses, and the peaks decay with the total decay rate of the exciton $k_{\mathrm{tot,X}}$. Here, the flat background can be both due to background photon–exciton photon pairs or delayed emission. The biexciton efficiency η_{BX} is equal to the amplitude ratio $A_0/A_{\pm 1}$.

The intensity-correlation function of the OFF state $g^{(2)}_{\mathrm{OFF}}$ contains fewer photon pairs due to a low photon-detection rate and is therefore noisy. Additionally, mixing in the ON state due to flickering introduces a slow component with a small amplitude which makes fitting a set of double-sided exponentials difficult. Therefore, we numerically integrate the intensity-correlation function to obtain the total number of photon pairs in the side peaks $a_{\pm 1}$ and the zero-delay peak a_0. We integrate all photon pairs within $|\tau \pm T| < 10$ ns for $a_{\pm 1}$ and within $|\tau| < 10$ ns for a_0 (Figure S3a). The integration range is limited to ± 10 ns around the peaks to minimize the contribution of the ON state to the total number of photon pairs.

There can be multiple sources of background that contribute to a_0 and $a_{\pm 1}$. Photons leaking into our experimental setup contribute to *uncorrelated* background which gives a *flat* background b in the intensity-correlation function. Since the exciton photon-detection rate n_{X} is much higher than the photon-detection rate of uncorrelated background $n_{\mathrm{f,bg}}$, the largest contribution to the intensity-correlation function is via background–exciton photon pairs. We estimate the contribution of the uncorrelated background b to $a_{\pm 1}$ and a_0 by integrating over a 10-ns delay-time range on the flat part of the correlation function ($|\tau \pm 200| < 10$ ns, where b is the average of the two), where the contribution of exciton–exciton and biexciton–exciton photon pairs is negligible (Figure S3a). Additionally, *correlated* background photons from excited-state decay of (organic molecules in) the glass substrate contribute to a *pulsed* background c. We characterize the pulsed background by measuring the substrate using nominally the same laser fluence as all single-QD measurements. Figure S3b shows the experimental decay curve of this measurement, where we observe very fast decay on top of a flat background. We extract a pulsed background photon-detection rate of $n_{\mathrm{p,bg}} = 4 \times 10^{-5}$ per laser pulse. Since the exciton photon-detection rate of $n_{\mathrm{X}} \approx 2 \times 10^{-2}$ per pulse is much higher than the photon-detection rate of correlated background $n_{\mathrm{p,bg}}$, the largest contribution to the intensity-correlation function is via background–exciton photon pairs. The pulsed background c, using the extracted photon-detection rate of the pulsed background, is given by:

$$c = \frac{1}{2}N_{\mathrm{p}}n_{\mathrm{X}}n_{\mathrm{p,bg}}, \tag{7}$$

where N_{p} is the total number of laser pulses in the experimental time of the time-gated decay curve, and the factor 1/2 accounts for the probability of background and exciton photons ending up on the same

detector (which are missed because of the detector dead time or because of the cross-correlation analysis procedure). Finally, we determine the biexciton-to-exciton efficiency ratio in the OFF state using

$$\frac{\eta_{\mathrm{BX}}}{\eta_{\mathrm{X}}} = \frac{2(a_0 - b - c)}{a_1 + a_{-1} - 2b - 2c}. \tag{8}$$

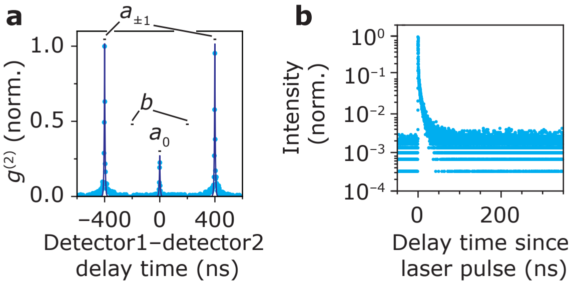

Figure S4 | Influence of substrate emission on the intensity-correlation function of the OFF state. **(a)** Intensity-correlation function of the OFF state $g^{(2)}_{\mathrm{OFF}}$ (QD A) indicating the integration ranges for the number of photon pairs in side peaks, zero-delay peak and the uncorrelated background **(b)** Experimental decay curve of the substrate, showing fast decaying emission, presumably from organic molecules in the glass substrate. The fast decaying emission contributes to a pulsed background c in $a_{\pm1}$ and a_0 via background–exciton photon pairs.

S3.2.2 Excited-state decay of (quenched) biexcitons

In this work, we analyze cascaded decay curves to extract excited-state decay rates. Most experimental cascaded decay curves contain no more than several hundred photon counts and are therefore noisy. It has been shown that *maximum-likelihood estimation* provides fitting parameters closer to the ground truth compared to *least-squares fitting* procedures, especially in the regime of low photon counts.[S8] Here, we implement a maximum-likelihood procedure on our decay curves with photon counts y_i at delay times t_i, assuming counting noise on the number of photon counts. We select a limited number of bins from the decay curve within $t \in (-5\text{ ns}, t_{\mathrm{m}})$, where t_{m} is manually chosen per single-QD measurement to neglect the slow component in the fitting procedure. We construct the maximum-likelihood estimator

$$\mathcal{L} = \prod_i P(A, k \,|\, t_i, y_i), \tag{9}$$

where $P = \mathrm{e}^{-\overline{y}}\overline{y}^{y_i}/y_i!$ is the probability to measure y_i photon counts at a delay time t_i for an expectation value $\overline{y}$. For the mean-value $\overline{y}$ at the delay time t_i we use eq 3, where the offset Δ and width σ found in Section S3.1 are used and the background B is found by averaging over the delay times $t \in (-5, -1)$ ns. In our fitting procedure, we minimize the log-likelihood function $-\log \mathcal{L}$ to find the decay-curve amplitude A and decay rate k.

S4 Perturbed electron wave functions due to electrostatic interactions

In the main text, we find a fast nonradiative recombination rate of the quenched biexciton BX* compared to the quenched exciton X* (Figure 3). In the conventional mechanism of trap-assisted recombination introduced by Yuan *et al.*, this means that the electron-trapping rate must be at least one order of magnitude higher for the biexciton state.[S9] In principle, the electron-trapping rate can be boosted in the biexciton state if the wave-function overlap with the trap state increases compared to the exciton. Here, we investigate whether electrostatic interactions in the biexciton state can significantly distort the electron wave functions and cause increased overlap with surface traps.

We construct the exciton and biexciton wave functions in core–shell QDs using a variational approach. It uses the particle-in-a-spherical-box model for the electron and hole levels and includes Coulomb interactions between the carriers. We assume that holes are confined to the CdSe core of radius a and that electrons are delocalized over the CdSe core and CdS shell (QD radius b) with no potential step between the core and shell.

We write the (bi)exciton state as a sum of single-particle product wave functions:

$$\Psi_{\mathrm{X}}(\mathbf{r}_{\mathrm{e}}, \mathbf{r}_{\mathrm{h}}) = \sum_i c_{\mathrm{X},i} \Psi^i(\mathbf{r}_{\mathrm{e}}, \mathbf{r}_{\mathrm{h}}), \tag{10a}$$

$$\Psi_{\mathrm{BX}}(\mathbf{r}_{\mathrm{e1}}, \mathbf{r}_{\mathrm{e2}}, \mathbf{r}_{\mathrm{h1}}, \mathbf{r}_{\mathrm{h2}}) = \sum_i c_{\mathrm{BX},i} \Psi^i(\mathbf{r}_{\mathrm{e1}}, \mathbf{r}_{\mathrm{e2}}, \mathbf{r}_{\mathrm{h1}}, \mathbf{r}_{\mathrm{h2}}) \tag{10b}$$

where only indistinguishable anti-symmetric states with paired electron and paired hole spins are relevant for our corrections to the wave function of the (bi)exciton ground state. The summations i run over all possible (bi)exciton states that can be constructed with the basis set of single-particle levels. To prevent the calculations from becoming too involved, we use a limited basis set for the electron including $n_{\mathrm{max}} = 3$ and $l_{\mathrm{max}} = 1$ and we fix the hole(s) in the 1S ground state. We find the coefficients $\mathbf{c}_{\mathrm{X,BX}}$ by minimizing the (bi)exciton energy

$$E_{\mathrm{X,BX}} = \langle \Psi_{\mathrm{X,BX}} | H_{\mathrm{X,BX}} | \Psi_{\mathrm{X,BX}} \rangle. \tag{11}$$

We optimize the electron wave function for the (bi)exciton including their potential energy in the potential landscape of the hole(s) in the 1S ground state. We find the electrostatic potential due to attraction by the hole ϕ_{h} by solving Poisson's equation:

$$\frac{2}{r}\frac{\mathrm{d}\phi_{\mathrm{h}}}{\mathrm{d}r} + \frac{\mathrm{d}^2\phi_{\mathrm{h}}}{\mathrm{d}r^2} = -\frac{qe}{\epsilon\epsilon_0}|\psi_{1\mathrm{S}}(r)|^2 \tag{12}$$

for the biexciton ($q = 2$) and the exciton ($q = 1$), where ϵ_0 is the vacuum permittivity and e the elementary charge. Note that the hole wave function is zero outside the CdSe core, which makes the hole potential a piece-wise function over the QD volume. We find the (bi)exciton energies using the Hamiltonians:

$$H_{\mathrm{X}} = H_0^{\mathrm{e}}(\mathbf{r}_{\mathrm{e}}) + H_0^{\mathrm{h}}(\mathbf{r}_{\mathrm{h}}) + V_{\mathrm{e}}(\mathbf{r}_{\mathrm{e}}) \tag{13a}$$

$$\begin{aligned} H_{\mathrm{BX}} = {} & H_0^{\mathrm{e1}}(\mathbf{r}_{\mathrm{e1}}) + H_0^{\mathrm{e2}}(\mathbf{r}_{\mathrm{e2}}) + H_0^{\mathrm{h1}}(\mathbf{r}_{\mathrm{h1}}) + H_0^{\mathrm{h2}}(\mathbf{r}_{\mathrm{h2}}) \\ & + V_{\mathrm{e1}}(\mathbf{r}_{\mathrm{e1}}) + V_{\mathrm{e2}}(\mathbf{r}_{\mathrm{e2}}) + V_{\mathrm{ee}}(\mathbf{r}_{\mathrm{e1}}, \mathbf{r}_{\mathrm{e2}}) + V_{\mathrm{hh}}(\mathbf{r}_{\mathrm{h1}}, \mathbf{r}_{\mathrm{h2}}), \end{aligned} \tag{13b}$$

where $H_0^{\mathrm{e,h}}$ give the energies of the unperturbed particle-in-a-spherical-box states, V_{ii} is the Coulomb operator for repulsion between pairs of like carriers, and V_i is the potential energy of an electron in the electrostatic potential of the hole(s). Here, we use $m_{\mathrm{h}} = 1.0m_0$ and $m_{\mathrm{e}} = 0.1m_0$ (with m_0 the electron rest mass in vacuum) for the effective hole and electron masses in CdSe and we use a homogeneous dielectric constant $\epsilon = 10$.[S10]

S4.1 Electron wave functions in a core–shell QD

We calculate the perturbed electron wave functions of the (bi)exciton in a core–shell QD with a core radius $a = 2$ nm and a QD radius $b = 4$ nm (Figure S4a,b). Figure S4a,b shows the optimized electron density of the (bi)exciton. We observe that the electron density of the biexciton is more confined to the QD core because of double the electrostatic attractions compared to the exciton. To quantify the perturbed probability distribution for the electron in the exciton and the biexciton, we plot the biexciton-to-exciton charge-density ratio along the x-axis in Figure S4c. To first order, the electron-trapping rate is proportional to the probability density of the electron at the position of the trap state. We observe that the charge-density ratio does not increase more than 25% from unity anywhere in the QD volume. Therefore we conclude that—whatever the actual position of the trap state—electrostatic interactions cannot explain the boost of the apparent electron-trapping rate in the biexciton.

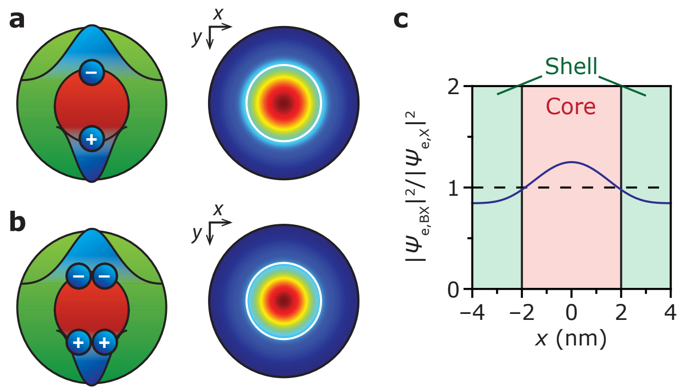

Figure S5 | Perturbed electron wave functions for a core–shell QD. (a) Exciton state for type-I½ semiconductors where the electron occupies the entire QD volume and the hole occupies the core only. **(b)** Same as **a**, but for the biexciton state. The electron density of the biexciton contracts slightly compared to the exciton, due to enhanced electrostatic attractions. **(c)** The biexciton-to-exciton charge-density ratio for the electron. The charge-density ratio does not change more than 25% from unity, which makes it unlikely that the electron-trapping rate is boosted significantly in the biexciton due to enhanced overlap with the trap state.

S4.2 Electron wave functions in a core–shell QD with a displaced core

A displaced core polarizes the (bi)exciton and electrostatic attractions pull the electron density towards the core and therefore closer to the QD surface (Figure S5a,b). Here, we investigate this effect quantitatively by displacing the CdSe core (a = 2 nm) 1 nm along the x-axis, closer to the QD surface (QD radius b = 4 nm). Figure S5a,b shows the optimized electron density of the (bi)exciton. We observe that the electron density of the biexciton is pulled closer to the QD surface than the exciton, due to enhanced electrostatic attractions. To quantify the perturbed probability distribution for the electron of the exciton and the biexciton, we plot the biexciton-to-exciton charge-density ratio along the x-axis (red) and the y-axis (blue) in Figure S4c. We observe that the charge-density ratio does not increase more than 30% from unity. Therefore, we conclude that also a displaced core cannot explain a boosted electron-trapping rate in the biexciton.

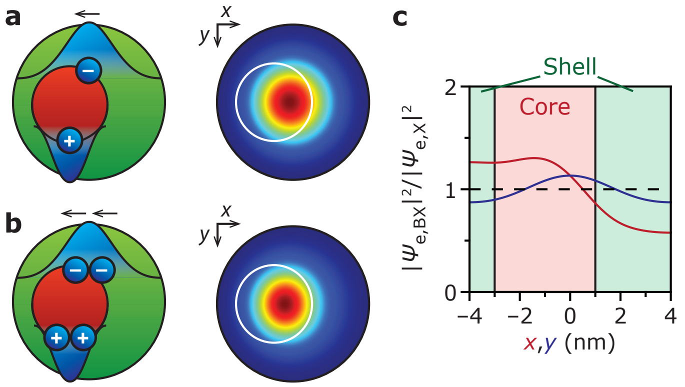

Figure S6 | Perturbed electron wave functions in a core–shell QD with a displaced core. (a) Exciton state for type-I½ semiconductors where the electron occupies the entire QD volume and the hole occupies the core. The electron density is pulled slightly towards the QD core. **(b)** Same as **a**, but for the biexciton state. Here, including both attractions and repulsions between all charge carriers, the electron density is pulled more towards the QD core compared to the exciton. **(c)** Line trace of the biexciton-to-exciton charge-density ratio for the electron along the x-axis (red) and the y-axis (blue). The charge-density ratio does not increase more than 30% from unity, which makes a boosted electron-trapping rate in the biexciton state unlikely.

S5 Trap-assisted recombination revisited

Our experimental results on the quenched (bi)exciton are inconsistent with the proposed trap-assisted recombination introduced by Yuan *et al.* where a *single* trapping process is followed up by nonradiative decay to the ground state. To match the experimental efficiencies, the trapping process for the quenched biexciton must be an order of magnitude faster, which cannot be explained by the expansion/contraction of the wave functions due to electrostatic interactions (Supporting Information section S4). Therefore, we propose an alternative model (Figure S6a,b) where the QD can cycle between a state with delocalized charge carriers (X′ or BX′) and charge-separated state with a trapped carrier (X″ or BX″). Very fast trapping k_t and detrapping k_{dt} compared to radiative k_r and nonradiative decay k_{nr} leads to a quasi-steady-state population ratio of the delocalized and charge-separated state. As a consequence, the quenched exciton X* and quenched biexciton BX* decay radiatively or nonradiatively through effective decay rates that are population-weighted averages of those of the delocalized states (X′ and BX′) and charge-separated states (X″ and BX″):

$$I_{X^*}(t) = P'_{X^*}k_{r,X'}\exp\Big[-\Big(P'_{X^*}k_{r,X'} + P''_{X^*}k_{nr,X''}\Big)t\Big] \tag{14a}$$

$$I_{BX^*}(t) = (P'_{BX^*}k_{r,BX'} + P''_{BX^*}k_{r,BX''})\exp\Big[-\Big(P'_{BX^*}k_{tot,BX'} + P''_{BX^*}k_{tot,BX''}\Big)t\Big]. \tag{14b}$$

Here, P' and P'' are the quasi-steady-state probabilities to be in the delocalized $[P' = k_{dt}(k_t + k_{dt})^{-1}]$ or charge-separated $[P'' = k_t(k_t + k_{dt})^{-1}]$ state. The effective radiative and nonradiative rates of the quenched (bi)exciton are weighted averages:

$$k_{r,X^*} = P'_{X^*}k_{r,X'} \tag{15a}$$

$$k_{nr,X^*} = P''_{X^*}k_{nr,X''} \tag{15b}$$

$$k_{r,BX^*} = P'_{BX^*}k_{r,BX'} + P''_{BX^*}k_{r,BX''} \tag{15c}$$

$$k_{nr,BX^*} = P'_{BX^*}k_{nr,BX'} + P''_{BX^*}k_{nr,BX''}. \tag{15d}$$

In the simplest model, blinking involves solely the opening and closing of a trap state. Opening of the trap state does not affect the delocalized exciton states (such as the radiative decay rates or any direct nonradiative recombination rate) apart from introducing the possibility of trapping. We thus assume that the radiative and nonradiative decay rates of the delocalized states X′ and BX′ are equal to the rates of the ON states X and BX. In the following calculations, we will use the extracted rates for the ON state of QD B from the main text. Figure S6c,d shows the effective radiative and nonradiative decay rates of the quenched (bi)exciton (eq 15) as a function of $k_t k_{dt}^{-1}$. In the limit of very small trapping/detrapping ratio $k_t k_{dt}^{-1} \to 0$, the population of the quenched (bi)exciton shifts to the delocalized state $P' = 1$. On the other hand, very large trapping/detrapping ratio $k_t k_{dt}^{-1} \to \infty$ drives the population towards the charge-separated state $P'' = 1$. Here, we assumed a non-emissive charge-separated exciton X″ ($k_{r,X''} = 0$ ns^{-1} and $k_{nr,X''} = 2$ ns^{-1}) and a highly quenched charge-separated biexciton ($k_{r,BX''} = 0.08$ ns^{-1} and $k_{nr,BX''} = 10$ ns^{-1}) with half the radiative decay rate of the regular biexciton.
From the integrated decay curves (eq 14), we obtain the efficiencies of the quenched exciton X* and biexciton BX* given by:

$$\eta_{X^*} = \frac{P'_{X^*}k_{r,X'}}{P'_{X^*}k_{r,X'} + P''_{X^*}k_{nr,X''}} \tag{16a}$$

$$\eta_{BX^*} = \frac{P'_{BX^*}k_{r,BX'} + P''_{BX^*}k_{r,BX''}}{P'_{BX^*}k_{tot,BX'} + P''_{BX^*}k_{tot,BX''}}. \tag{16b}$$

Note that the efficiencies are not the population-weighted average efficiencies of the delocalized and charge-separated states, but the ratio of the population-weighted averages of radiative and total decay rates. Figure S6e shows the quenched exciton X* efficiency (color coded) as a function of the trapping/detrapping ratio k_t/k_{dt} and the nonradiative decay rate of the charge-separated state $k_{nr,X''}$.

Qualitatively we observe that the efficiency of the quenched exciton is high (red color code, bottom left) if (1) the nonradiative rate of the charge-separated state is low and (2) the trapping/detrapping ratio is low (high population of delocalized exciton). On the contrary, if both the nonradiative rate and the trapping/detrapping ratio are high the efficiency is low (blue color code). Interestingly, we find that multiple combinations of parameter values—nonradiative decay rate and trapping/detrapping ratio—are consistent with the experimentally observed efficiency of the quenched exciton ($\eta_{X^*} = 6.5\%$, Figure 1g). Figure S6e shows the iso-efficiency line (white) giving all combinations of nonradiative decay rate and trapping/detrapping ratio consistent with the quenched-exciton efficiency. From the amplitude ratio of the time-averaged decay curves of QD B (Figure 1g), we observe that the effective radiative rate of the quenched exciton X^* is practically equal to the radiative rate of the regular exciton X'. This means near-unity population of the regular exciton state $P' = 1$ for QD B in the OFF state and $k_t/k_{dt} \ll 1$. Low population of the charge-separated state means that the nonradiative decay rate of this state must be very high ($k_{nr,X''} > 10^2$) to yield an effective efficiency of only 6.5%, but determining an exact value is impossible. Figure S6f shows the same calculations for the quenched biexciton, which are qualitatively very similar.

In the QD sample studied in this work, most QDs that showed trap-assisted recombination in the OFF state (QDs 5–10 in Supporting Information S6) had near-unity population of the delocalized exciton state ($A_{OFF}/A_{ON} \approx 1$). In Figure S7 we show the time-averaged decay curves of single QDs from the same synthesis batch that have comparable population of the delocalized and charge-separated state.

As discussed above, it is very difficult to quantify the nonradiative decay rates of the charge-separated states, but we do know that the nonradiative decay rate of the charge-separated state is much faster than the radiative decay rate. We can still learn something about the charge-separated states from the parameter boost factor K introduced in the main text: the excess nonradiative decay rate of the quenched biexciton relative to the nonradiative decay rate of the quenched exciton. Following the revisited model for trap-assisted recombination, we find

$$K = \frac{k_{nr,BX^*} - k_{nr,BX}}{k_{nr,X^*}} = \frac{P'_{BX^*} k_{nr,BX'} + P''_{BX^*} k_{nr,BX''} - k_{nr,BX'}}{P''_{X^*} k_{nr,X''}} \approx 2 \frac{k_{nr,BX''}}{k_{nr,X''}}, \tag{17}$$

where we used that $P'_{BX^*} \approx 1$ in the limit of very small trapping/detrapping ratios, and $P''_{BX^*}/P''_{X^*} \approx 2$ if we assume statistical scaling of the electron-trapping rate. This allows us to relate the boost factor K to relative nonradiative rate of the charge-separated states. Plugging in the experimental boost factor of QD B, $K = 14.7$, we find that $k_{nr,BX''}/k_{nr,X''} \approx 7$. This shows that the nonradiative decay rate of the charge-separated biexciton BX'' is much faster than the Auger recombination rate in the regular biexciton BX'. As discussed in the main text, this can be explained by considering that the localized charge carrier of the charge-separated biexciton BX'' is an efficient Auger acceptor.

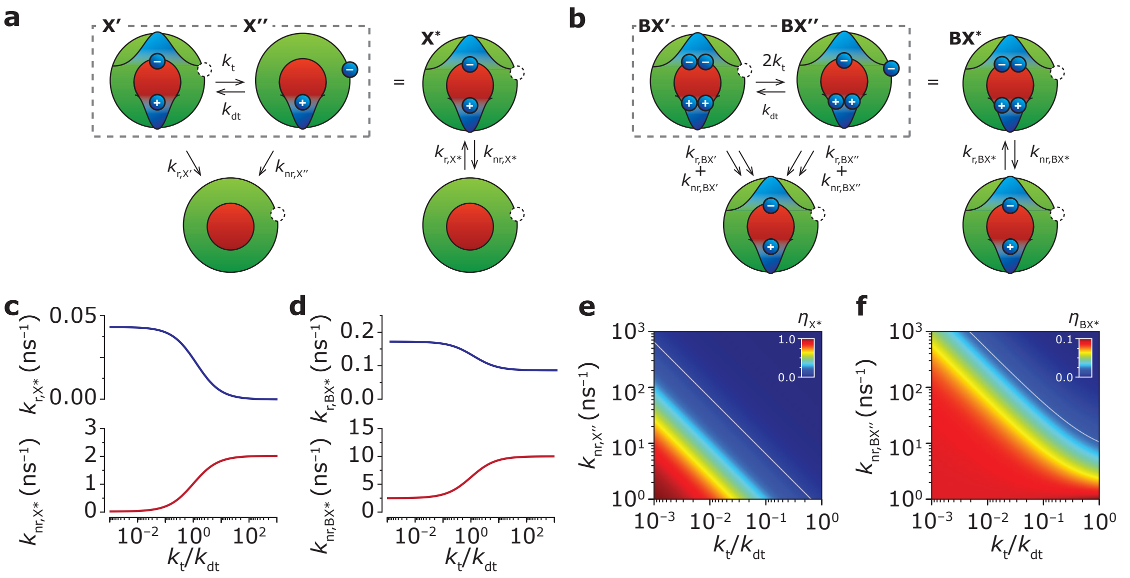

Figure S7 | Quenching mechanism including ultrafast switching. **(a)** Reversible switching between a delocalized exciton X′ and charge-separated exciton X″ as a model for the quenched exciton X*. **(b)** Same as **a**, but for the biexciton. **(c)** Effective radiative k_{r,X^*} and nonradiative decay rates k_{nr,X^*} of the quenched exciton X* as a function of the trapping/detrapping ratio $k_t k_{dt}^{-1}$. The quenched exciton X* gradually switches from the delocalized exciton X′ to the charge-separated exciton X″ going from low to high trapping/detrapping ratio $k_t k_{dt}^{-1}$. **(d)** Same as **c**, but for the quenched biexciton BX*. **(e)** Quenched exciton efficiency η_{X^*} (color coded) as a function of the trapping/detrapping ratio and the. The white line is the iso-efficiency line for the single-QD measurement in the main text ($\eta_{X^*} = 6.5\%$), giving the nonradiative decay rate of the charge-separated exciton $k_{nr,X''}$ as a function of the trapping/detrapping ratio. **(f)** Same as **e**, but for the quenched biexciton.

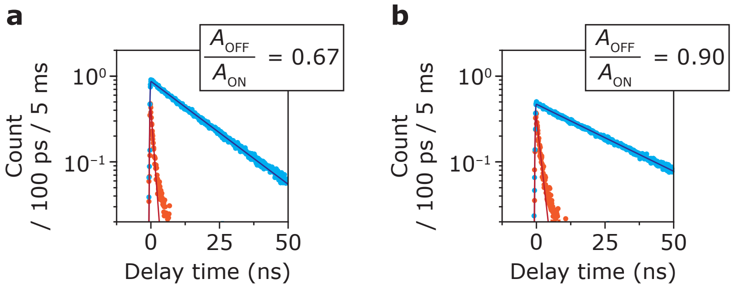

Figure S8 | Quantum dots with a comparable quasi-steady-state population of delocalized and charge-separated exciton states. **(a)** Time-averaged decay curves of a single QD from the same synthesis batch as QD A and QD B in the main text and the QDs in the extended data Section S6, in the ON (blue) and OFF (red) state. From the amplitude ratio $A_{OFF}/A_{ON} = 0.5$ we quantify a trapping/detrapping ratio $k_t k_{dt}^{-1} \approx 2$, which means comparable population of delocalized and charge-separated exciton states. **(b)** Same as **a**, but for a different QD from the same synthesis batch. Here, the amplitude ratio $A_{OFF}/A_{ON} = 0.9$ corresponds to a trapping/detrapping ratio $k_t k_{dt}^{-1} = 0.1$.

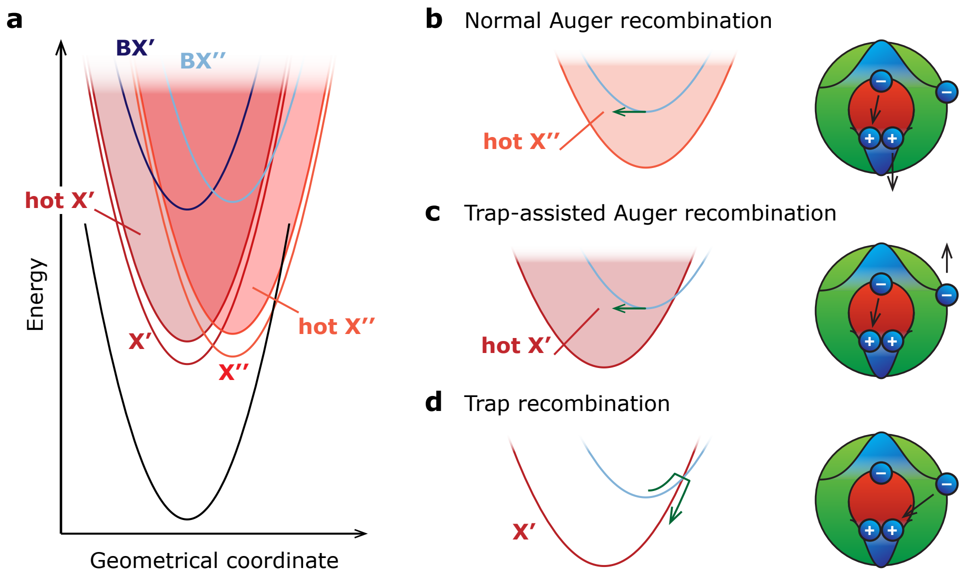

Figure S9 | Quenching mechanisms of the charged-separated biexciton BX″. **(a)** Configuration-coordinate diagram of the ground-state delocalized exciton X′ (dark red line) and the hot delocalized excitons (dark red shading), the ground-state charge-separated exciton X″ (light red line) and the hot charge-separated excitons (light red shading), ground-state delocalized biexciton BX′ (dark blue), and the ground-state charge-separated biexciton BX″ (light blue). The hot biexciton states are not shown because they do not contribute to the decay pathways of the biexciton and/or exciton states. (b) Conventional Auger recombination of the charge-separated biexciton BX″ following the positive-trion pathway leading to a hot charge-separated exciton state X″, i.e. the final state constitutes a localized electron and a hot hole. (c) Trap-assisted Auger recombination where the localized charge carrier acts as an Auger acceptor leading to a hot delocalized exciton state X′, i.e. the remaining hole is in the 1S ground level while the excited electron occupies a high-energy level. (d) Direct trap recombination of the charge-separated biexciton leading to a delocalized exciton state X′, i.e. both the remaining electron and hole occupy an 1S ground level but the QD is in a high vibrational state.

S6 Extended data

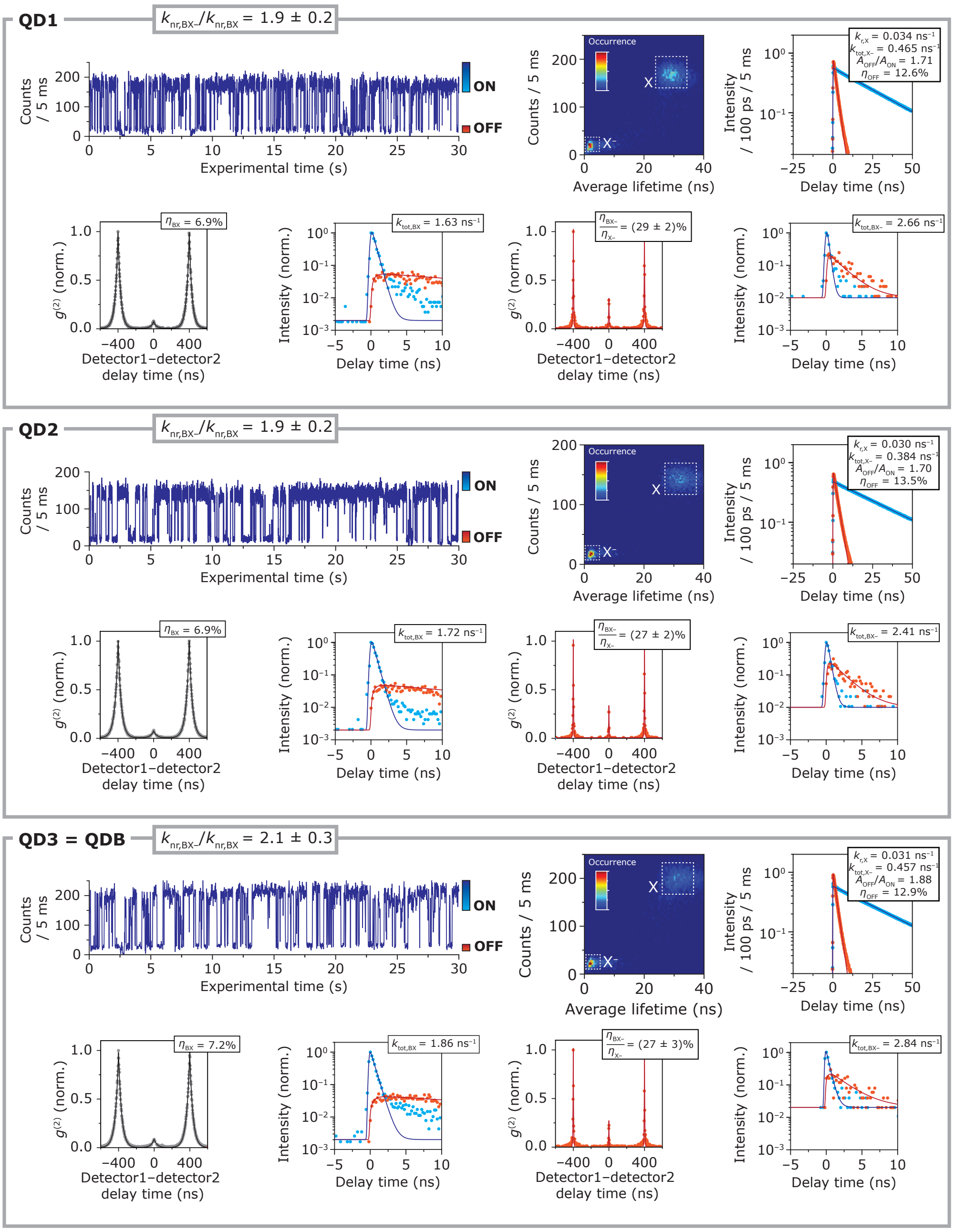

Figure S10 | For single QDs 1–3, showing intermittent charging/discharging: Top left; blinking trace with 5-ms bins of selected experimental time range of 30 seconds. Top right; Fluorescence-lifetime–intensity distribution and time-averaged decay curves. Bottom row from left to right; intensity-correlation function of the ON state, cascaded decay curves of first (blue) and second (red) of the biexciton cascade in the ON state, intensity-correlation function of the OFF state, and cascaded decay curves of first (blue) and second (red) of the biexciton cascade in the OFF state.

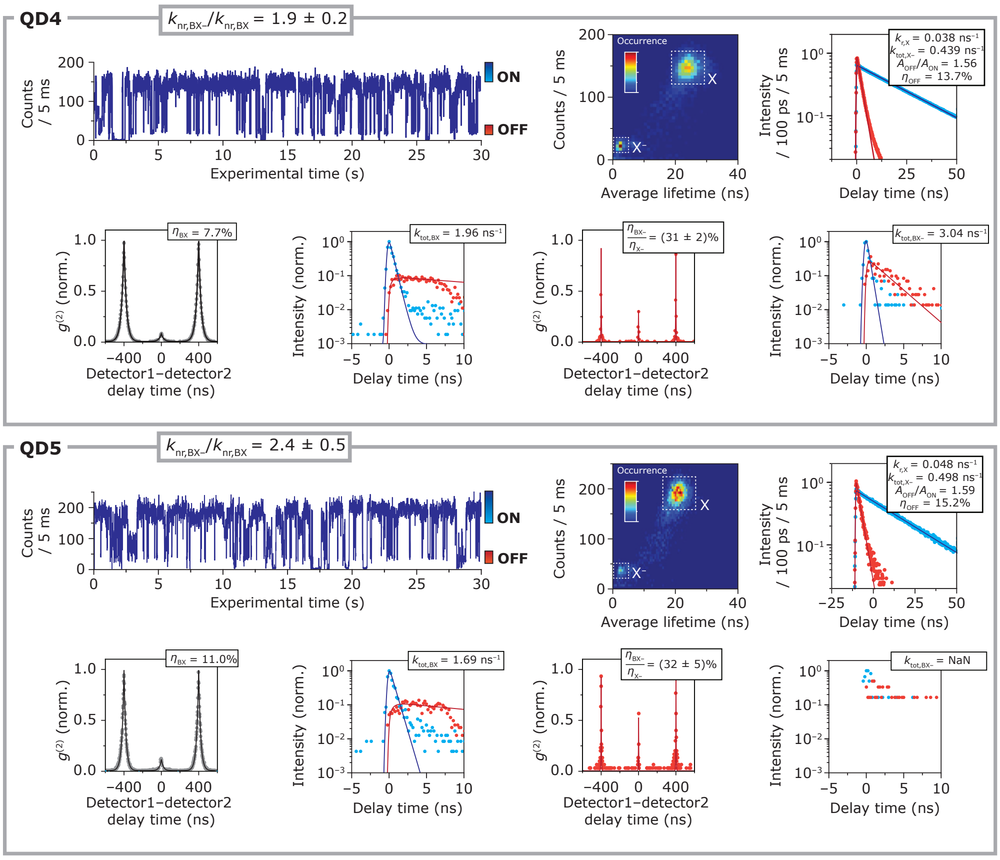

Figure S11 | For single QDs 4 and 5, showing intermittent charging/discharging: Top left; blinking trace with 5-ms bins of selected experimental time range of 30 seconds. Top right; Fluorescence-lifetime–intensity distribution and time-averaged decay curves. Bottom row from left to right; intensity-correlation function of the ON state, cascaded decay curves of first (blue) and second (red) of the biexciton cascade in the ON state, intensity-correlation function of the OFF state, and cascaded decay curves of first (blue) and second (red) of the biexciton cascade in the OFF state.

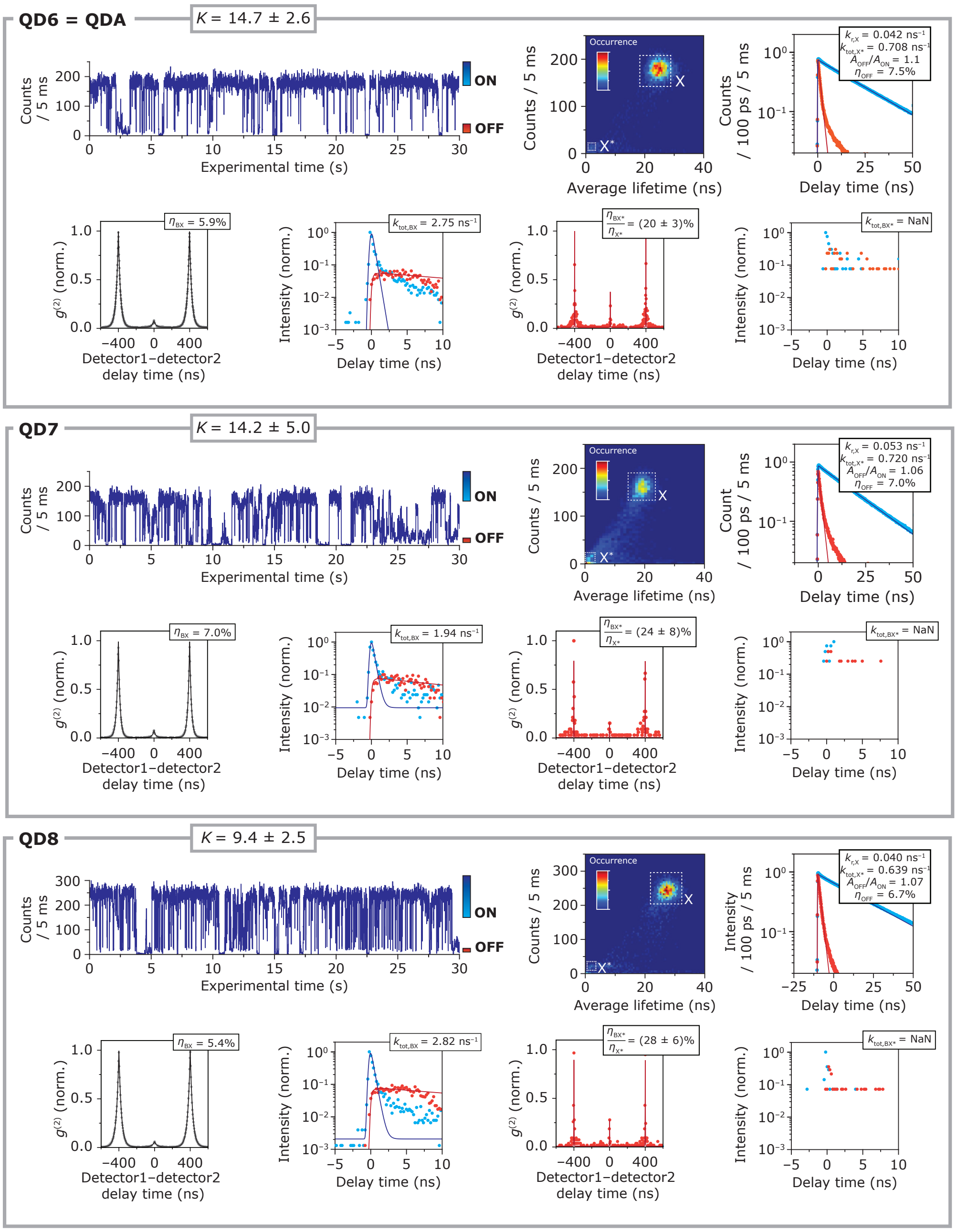

Figure S12 | For single QDs 6–8, showing intermittent opening/closing of traps: Top left; blinking trace with 5-ms bins of selected experimental time range of 30 seconds. Top right; Fluorescence-lifetime–intensity distribution and time-averaged decay curves. Bottom row from left to right; intensity-correlation function of the ON state, cascaded decay curves of first (blue) and second (red) of the biexciton cascade in the ON state, intensity-correlation function of the OFF state, and cascaded decay curves of first (blue) and second (red) of the biexciton cascade in the OFF state.

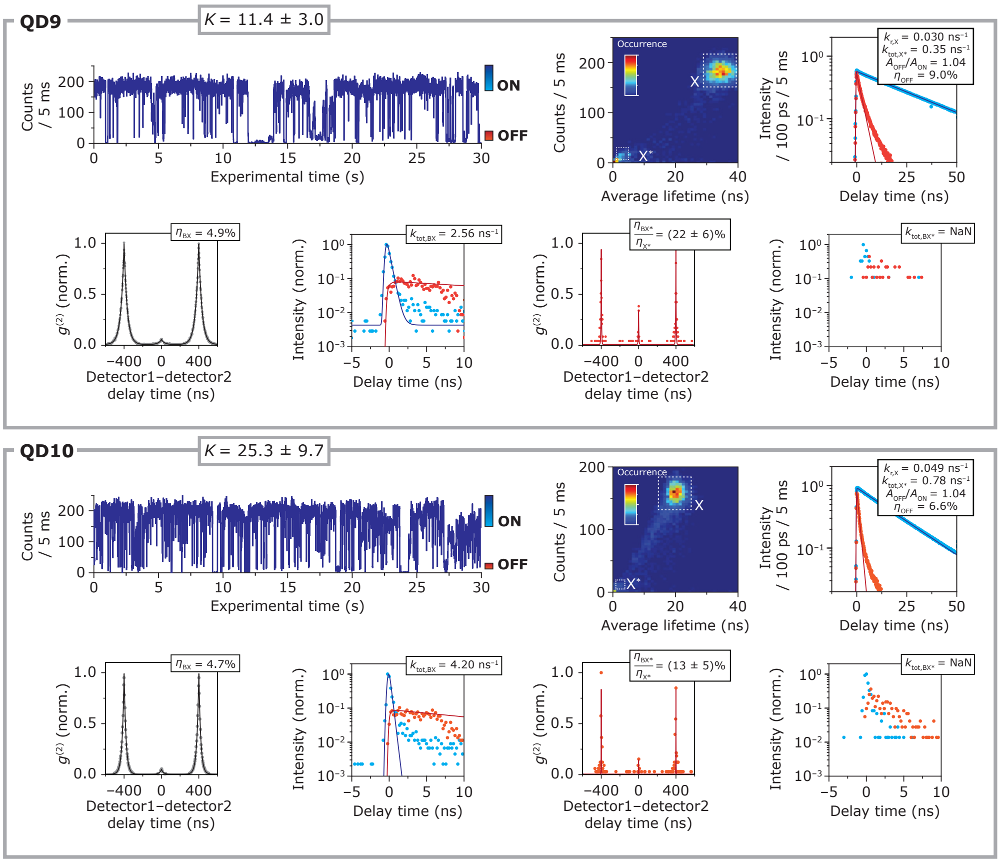

Figure S13 | For single QDs 9 and 10, showing intermittent opening/closing of traps: Top left; blinking trace with 5-ms bins of selected experimental time range of 30 seconds. Top right; Fluorescence-lifetime–intensity distribution and time-averaged decay curves. Bottom row from left to right; intensity-correlation function of the ON state, cascaded decay curves of first (blue) and second (red) of the biexciton cascade in the ON state, intensity-correlation function of the OFF state, and cascaded decay curves of first (blue) and second (red) of the biexciton cascade in the OFF state.

Supporting References

(S1) Chen, O.; Zhao, J.; Chauhan, V. P.; Cui, J.; Wong, C.; Harris, D.K.; Wei, H.; Han, H.S.; Fukumura, D.; Jain, R.K.; Bawendi, M.G. Compact High-Quality CdSe–CdS Core–Shell Nanocrystals with Narrow Emission Linewidths and Suppressed Blinking, *Nat. Mater.* **2013**, *12*, 445–451.

(S2) Boldt, K.; Kirkwood, N.; Beane, G.A.; Mulvaney, P. Synthesis of Highly Luminescent and Photo-stable, Graded Shell CdSe/$Cd_xZn_{1-x}S$ Nanoparticles by In Situ Alloying, *Chem. Mater.* **2013**, *25*, 4731–4738.

(S3) Hanifi, D.A.; Bronstein, N.D.; Koscher, B.A.; Nett, Z.; Swabeck, J.K.; Takano, K.; Schwartzberg, A.M.; Maserati, L.; Vandewal, K.; van de Burgt, Y.; Salleo, A., Alivisatos, P.A. Redefining Near-Unity Luminescence in Quantum Dots with Photothermal Threshold Quantum Yield, *Science* **2019**, *363*, 199–1202.

(S4) Jasieniak, J.; Smith, L.; van Embden, J.; Mulvaney, P. Re-examination of the Size-Dependent Absorption Properties of CdSe Quantum Dots, *J. Phys. Chem. C.* **2009**, *113*, 19468–19474.

(S5) Hinterding, S.O.M; Rabouw, F.T. PHoton Arrival-Time STudio (PHAST), *Zenodo* **2020** https://doi.org/10.5281/zenodo.4354144.

(S6) Galland, C.; Yagnaseni, G.; Steinbrück, A.; Sykora, M.; Hollingsworth, J.A.; Klimov, V.I.; Htoon, H. Two Types of Luminescence Blinking Revealed by Spectroelectrochemistry of Single Quantum Dots, *Nature* **2011**, *479*, 203–207.

(S7) Brokmann, X.; Coolen, L.; Dahan, M.; Hermier, J.P. Measurement of the Radiative and Nonradiative Decay of Single CdSe Nanocrystals Through a Controlled Modification of their Spontaneous Emission, *Phys. Rev. Lett.* **2004**, *93*, 107403.

(S8) Maus, M.; Cotlet, M.; Hofkens, J.; Gensch, T.; de Schryver, F. An Experimental Eomparison of the Maximum Likelihood Estimation and Nonlinear Least-Squares Fluorescence Lifetime Analysis of Single Molecules, *Anal. Chem.* **2001**, *73*, 2078–2086.

(S9) Yuan, G.; Gomez, D.E.; Kirkwood, N.; Boldt, K.; Mulvaney, P. Two Mechanisms Determine Quantum Dot Blinking, *ACS Nano* **2018**, *12*, 3397–3405.

(S10) De Mello Donega, C. Synthesis and Properties of Colloidal Heteronanocrystals, *Chem. Soc. Rev.* **2011**, *40*, 1512–1546.
